# Molecular Rubies in Photoredox Catalysis

**DOI:** 10.3389/fchem.2022.887439

**Published:** 2022-04-07

**Authors:** Steven Sittel, Robert Naumann, Katja Heinze

**Affiliations:** Department of Chemistry, Johannes Gutenberg University, Mainz, Germany

**Keywords:** chromium, radical cation, Diels-Alder reaction, [4+2] cycloaddition, photoredox catalysis, singlet oxygen

## Abstract

The molecular ruby **[Cr(tpe)**_**2**_ **]** ^**3+**^ and the tris(bipyridine) chromium(III) complex **[Cr(dmcbpy)**_**3**_ **]** ^**3+**^ as well as the tris(bipyrazine)ruthenium(II) complex **[Ru(bpz)**_**3**_ **]** ^**2+**^ were employed in the visible light-induced radical cation [4+2] cycloaddition (tpe = 1,1,1-tris(pyrid-2-yl)ethane, dmcbpy = 4,4′-dimethoxycarbonyl-2,2′-bipyridine, bpz = 2,2′-bipyrazine), while **[Cr(ddpd)**_**2**_ **]** ^**3+**^ serves as a control system (ddpd = *N*,*N*′-dimethyl-*N*,*N*′-dipyridin-2-ylpyridine-2,6-diamine). Along with an updated mechanistic proposal for the Cr^III^ driven catalytic cycle based on redox chemistry, Stern-Volmer analyses, UV/Vis/NIR spectroscopic and nanosecond laser flash photolysis studies, we demonstrate that the very weakly absorbing photocatalyst **[Cr(tpe)**_**2**_ **]** ^**3+**^ outcompetes **[Cr(dmcbpy)**_**3**_ **]** ^**3+**^ and even **[Ru(bpz)**_**3**_ **]** ^**2+**^ in particular at low catalyst loadings, which appears contradictory at first sight. The high photostability, the reversible redoxchemistry and the very long excited state lifetime account for the exceptional performance and even reusability of **[Cr(tpe)**_**2**_ **]** ^**3+**^ in this photoredox catalytic system.

## Graphical Abstract

The molecular ruby **[Cr(tpe)**_**2**_ **]** ^**3+**^ catalyzes the visible light-induced radical cation [4+2] cycloaddition with unprecedented photostability and recyclability. Detailed mechanistic studies reveal that reductive quenching of **[Cr(tpe)**_**2**_ **]** ^**3+**^ is fast while the first C-C formation is the slowest step and triplet oxygen is required to rapidly regenerate **[Cr(tpe)**_**2**_ **]** ^**3+**^ and prevent detrimental back-electron transfer.
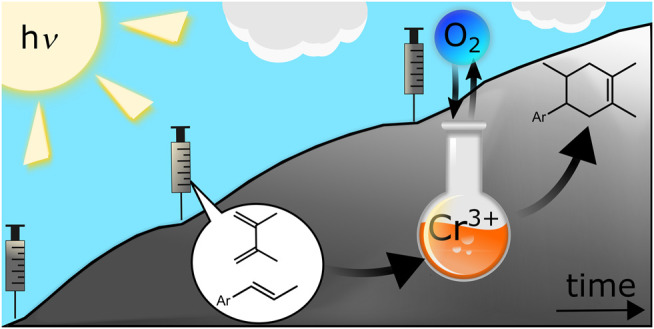

## Introduction

**SCHEME 1:**
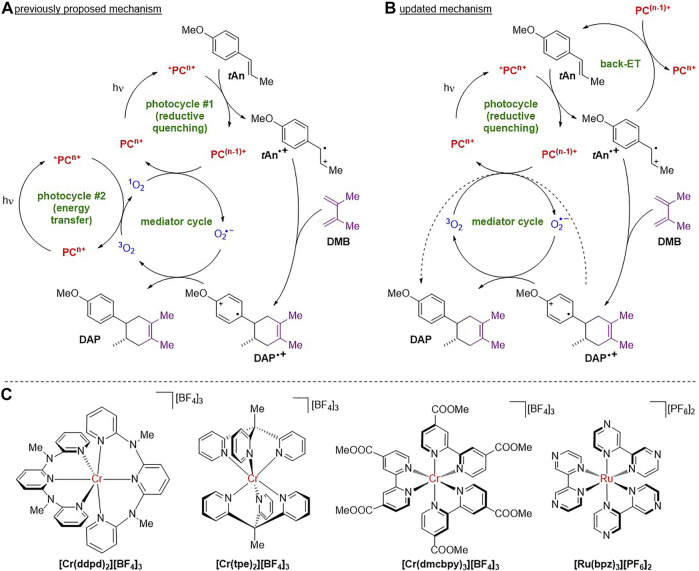
**(A)** Previously postulated mechanism of the visible light-driven radical cation [4+2] cycloaddition of *trans*-anethole (***t*An**) and 2,3-dimethyl-1,3-butadiene (**DMB**, displayed as *s*-*cis* conformer) with chromium(III) photocatalyst **PC** ^**3+**^ **[Cr(dmcbpy)**_**3**_ **]** ^**3+**^ (n = 3) to the Diels-Alder product **DAP**. **(B)** Updated outline of the catalytic cycle. **(C)** Chromium(III) and ruthenium(II) photocatalysts **PC** ^**n+**^ employed in this study.

Complexes with long-lived luminescent metal-to-ligand charge transfer (MLCT) excited states, namely Ru^II^, Ir^III^, Os^II^, or Re^I^ complexes with polypyridine ligands, are the workhorses of many photoredox catalytic cycles, luminescent devices, sensing applications and solar energy conversion processes (Twilton, et al., 2017; Yoon, et al., 2010; Magnuson, et al., 2009; Costa, et al., 2012; Baldo, et al., 2020 Hagfeld, et al., 2010; Heinemann et al., 2017; Mari, et al., 2015; Li, et al., 2018; Yam, et al., 2020). A huge interest exists in replacing the precious metal ions by cheaper, Earth-abundant metal ions such as chromium, iron and copper and considerable progress has been achieved in the last years (Wenger, 2018; Hockin, et al., 2019; Förster and Heinze, 2020; Büldt and Wenger, 2017; Otto, et al., 2018a; Scattergood, 2020; Lindh, et al., 2020; Wenger, 2021; Hossain, et al., 2019). Reductively initiated processes were successfully employed in several instances, e.g. using Mn^I^, Fe^II^ and Cu^I^ photocatalysts (Herr, et al., 2021; Leis, et al., 2022; Hossain, et al., 2019). Oxidative processes photoinduced by first row transition metal complexes are comparably scarce and have been reported with iron(III) and cobalt(III) (Aydogan, et al., 2021; Pal, et al., 2018) and in particular with chromium(III) photocatalysts (Stevenson, et al., 2015; Higgins, et al., 2016). Oxidatively induced photocatalytic processes, e.g. radical cation [4+2] cycloadditions, have been described by Yoon and coworkers using photooxidizing ruthenium(II) photocatalysts (Lin, et al., 2011; Farney, et al., 2019) and by Shores and others using highly photooxidizing Cr^III^ complexes with 2,2′-bipyridine or 9,10-phenanthroline ligands as photocatalysts **PC** ^**3+**^ (Scheme 1A) (Stevenson, et al., 2015; Higgins, et al., 2016; Stevenson, et al., 2017; Higgins, et al., 2018). For example, the highly oxidizing complex **[Cr**(**dmcbpy)**_**3**_ **]** ^**3+**^ (1 mol%, CH_3_NO_2_, 300–419 nm light, 24–48 h, dmcbpy = 4,4′-dimethoxycarbonyl-2,2′-bipyridine) catalyzes the Diels-Alder reaction of various dienes such as 2,3-dimethyl-1,3-butadiene (**DMB**) with styrenes such as *trans*-anethole (***t*An**) to the Diels-Alder product **DAP** (Scheme 1A) (Stevenson, et al., 2015) TiO_2_/LiClO_4_ also catalyzed this reaction using UV light (365 nm, Nakayama, et al., 2019). Radical cations ***t*An** ^**•+**^ and **DAP** ^**•+**^ are the proposed key intermediates in this radical cation [4+2] cycloaddition, but they were never observed in this context (Scheme 1A). The ***t*An** radical cation ***t*An** ^**•+**^ had been independently generated by UV light excitation through a biphotonic photoionization. The radical cation ***t*An** ^**•+**^ was observed by nanosecond laser flash photolysis (LFP, 308 nm or 266 nm excitation) by its excited state absorptions at 320–390 nm and 520–660 nm (Murphy, et al., 2000; Johnston and Schepp, 1993). The radical cation ***t*An** ^**•+**^ stabilized with [FeCl_4_]^–^ had even been isolated and an absorption band at 580 nm had been reported (Horibe, et al., 2019). Other methoxy-substituted aromatic radical cations prepared by oxidation with MoCl_5_ were successfully characterized by EPR spectroscopy (Leppin, et al., 2015; Schubert, et al., 2016). In the Cr^III^ or Ru^II^ photocatalyzed reactions, the radical cations ***t*An** ^**•+**^/**DAP** ^**•+**^ were not yet identified spectroscopically (Stevenson, et al., 2015; Higgins, et al., 2016; Lin, et al., 2011; Farney, et al., 2019).

For Cr^III^ photocatalysts and organic thioxanthylium and benzothiadiazole photocatalysts (Tanaka, et al., 2020; Huber, et al., 2020), oxygen is required for the regeneration of the reduced photocatalyst **PC** ^**(n−1)+**^ (Scheme 1A, mediator cycle). Interestingly, Rappé and Shores suggested that singlet oxygen ^1^O_2_ is required to regenerate the chromium(III)-based **PC** ^**3+**^ forming superoxide O_2_^•–^, which in turn reduces the cyclohexenyl radical **DAP** ^**•+**^ (Scheme 1A) (Higgins, et al., 2016) According to the suggested mechanism, ^1^O_2_ forms in a separate energy transfer photocycle (Scheme 1A, photocycle #2) so that two photons would be required for a single turnover. The requirement for the O_2_^•–^/^1^O_2_ couple instead of the conventional redoxcouple O_2_^•–^/^3^O_2_ was invoked by the negative redox potential of the latter (E_½_ = –1.29 V vs. ferrocene in CH_3_CN) (Singh and Evans, 2006), which would be insufficient for photocatalyst regeneration (Higgins, et al., 2016). In contrast to Cr^III^ photocatalysts requiring O_2_, ruthenium(II)-based photocatalysts seem to operate via a chain mechanism without the need for invoking ^1^O_2_ (Lin, et al., 2011) and even in the absence of oxygen (Farney, et al., 2019).

Under photolysis conditions, Cr^III^ and Ru^II^ complexes of bipyridine-type ligands are photolabile (Maestri, et al., 1976; Jamieson, et al., 1979; Constable, et al., 2014; Soupart, et al., 2020; Limburg, et al., 2016). Ligand loss is observed under irradiation, forming e.g., [(µ-OH)_2_(Cr(bpy)_2_)_2_]^4+^ (bpy = 2,2′-bipyridine) (Otto, et al., 2015; Otto, et al., 2018). Reduced complexes such as [Cr(Ph_2_phen)_3_]^2+^ furthermore were reported to decompose in the presence of O_2_ to Cr=O species (Ph_2_phen = 4,7-diphenyl-1,10-phenanthroline) (Higgins, et al., 2016). In contrast, Cr^III^ complexes with tridentate ligands, in particular those inducing a very strong ligand field by large bite angles, are highly photostable, often strongly luminescent with very high excited state lifetimes, and are hence called “molecular rubies” (Otto, et al., 2015; Otto, et al., 2018b; Jiménez, et al., 2019; Treiling, et al., 2019; Reichenauer, et al., 2021). The long-lived excited states of the molecular rubies are metal-centered spin-flip states (doublet states, ^2^E/^2^T_1_) in contrast to the typically exploited triplet MLCT excited states of Ru^II^ or Ir^III^ complexes (Kitzmann, et al., 2022). Important prerequisites for the high lifetime and the photostability of molecular rubies are the large ligand field splitting resulting in dissociative metal-centered quartet states (^4^T_2_) being shifted to high energy and fast intersystem crossing (ISC) processes that rapidly depopulate the ^4^T_2_ states to arrive at the long-lived ^2^E/^2^T_1_ states with high efficiency and without detrimental back-ISC processes (Otto, et al., 2018a; Kitzmann, et al., 2022). Potential disadvantages of the molecular rubies for photoredox applications are their comparably small extinction coefficients in the visible spectral region due to the Laporte-forbidden nature of the ^4^A_2_ → ^4^T_2_ excitation and the comparably low excited state energies (1.6–1.75 eV) (Otto, et al., 2018b; Kitzmann, et al., 2022).

In the present study we explore the Cr^III^ photocatalysts **[Cr**(**ddpd)**_**2**_ **]** ^**3+**^ and **[Cr**(**tpe)**_**2**_ **]** ^**3+**^ (Scheme 1B, ddpd = *N*,*N*′-dimethyl-*N*,*N*′-dipyridin-2-ylpyridine-2,6-diamine, tpe = 1,1,1-tris(pyrid-2-yl)ethane; Otto, et al., 2015; Treiling, et al., 2019) with the strong field ligands ddpd and tpe, respectively, as potential photostable photocatalysts **PC** ^**3+**^ for the visible light-driven radical cation [4+2] cycloaddition of ***t*An** and **DMB** (460 nm). We compare the performance with the reported optimized photocatalysts **[Cr**(**dmcbpy)**_**3**_ **]** ^**3+**^ and **[Ru**(**bpz)**_**3**_ **]** ^**2+**^ (bpz = 2,2′-bipyrazine; Crutchley, et al., 1980; Schultz, et al., 2015) and we investigate the role of oxygen for the regeneration of the Cr^III^ photocatalysts **PC** ^**3+**^ using nanosecond LFP studies to arrive at an updated mechanistic picture of the Cr^III^ photocatalyzed radical cation Diels-Alder cycloaddition (Scheme 1B). Finally, we discuss the relevance and importance of extinction coefficients and photostability of the photocatalysts **PC** ^**3+**^ and detrimental back-electron transfer processes laying the foundation for exploiting stable molecular rubies in forthcoming efficient photoredox reactions.

## Experimental Details

**General.** The photocatalysts **[Cr**(**ddpd)**_**2**_ **][BF**_**4**_ **]**_**3**_ (Otto, et al., 2015; Wang, et al., 2022), **[Cr**(**tpe)**_**2**_ **][BF**_**4**_ **]**_**3**_ (Treiling, et al., 2019) and **[Cr**(**dmcbpy)**_**3**_ **][BF**_**4**_ **]**_**3**_ (McDaniel, et al., 2010) were prepared according to literature procedures. 2,3-Dimethyl-1,3-butadiene (**DMB**, 98%, Sigma-Aldrich with 100 ppm 3,5-di-*tert*-butyl-4-hydroxytoluene as stabilizer) was distilled prior to use. **[Ru**(**bpz)**_**3**_ **][PF**_**6**_ **]**_**2**_ (≥95%, Sigma-Aldrich), *trans*-anethole (***t*An**, 99%, Sigma), naphthalene (99%, Acros), CH_3_CN for photoreactions (VWR Chemicals, HPLC grade), CH_3_CN for spectroscopy (Supelco, hypergrade) and CD_3_CN (98.5% D, Deutero) were commercially available and used as received.

UV/Vis absorption spectra were recorded on a *Jasco V770* spectrometer using conventional 1.0 cm quartz cells for studies under air and 1.0 cm quartz cells with a Schott valve for studies under inert atmosphere. Emission spectra and luminescence decay curves were recorded with a FLS1000 spectrometer from *Edinburgh Instruments* equipped with the cooled red and NIR sensitive photomultiplier detectors PMT-980 and N-G09 PMT-1700, together covering the spectral range between 200 and 1700 nm. A xenon arc lamp Xe2 (450 W) was used for excitation in steady-state measurements. Time-resolved luminescence experiments on microsecond to millisecond timescale were performed in the multi-channel scaling mode employing the μs-xenon-flashlamp µF2 (pulse width ca. 2 µs) as excitation source. Measurements on the nanosecond timescale were conducted in the TCSPC mode using a SUPERK FIANUM laser FIU-6 from *NKT Photonics* as excitation source. Absolute luminescence quantum yields *Φ* were determined using an integrating sphere from *Edinburgh Instruments*. Relative uncertainty of *Φ* is estimated to be ±10%. Stern-Volmer studies with oxygen were performed in a long-neck quartz cuvette sealed with a septum. After purging the sample solutions for about 30 min with Argon using a cannula, the emission decay was measured and the resulting lifetime was used as starting point for the Stern-Volmer analysis. To increase the oxygen partial pressures *p*_O2_ stepwise, air was added successively to the cuvette via a syringe until air-saturation is reached. After each addition, the solution was shaken for about 2 min. The corresponding *p*_O2_ has been determined employing the fiber-optic oxygen meter FireSting-O2 equipped with the solvent resistant probe tip OXSOLV-PT from *pyroscience*. The fiber of the probe is covered with a 150 mm cannula allowing it to pierce the septum without damaging the sensor. The sensor has been calibrated with deaerated and air-saturated solution. To assure that the *p*_O2_ is identical during the oxygen addition and the corresponding emission decay measurement, the emission decay was always recorded after the determination of *p*_O2_ as small amounts of air are brought into the cuvette with the cannula and are dissolved in the solution. Transient absorption spectroscopy experiments were conducted using a LP980KS setup from *Edinburgh Instruments* equipped with a Nano LG 300-10 Nd:YAG laser from *Litron* (ca. 5 ns pulse width). The frequency-tripled output with a wavelength of 355 nm was employed for the excitation (30 mJ pulse energy). To ensure homogeneous excitation of the whole observation volume (10 mm × 10 mm quartz cuvette) the beam was brought to a diameter of 1.2 cm using a beam expander. A pulsed Xe-lamp from *Hamamatsu* (150 W, XBO) was used as probe light source. The transient absorption spectra were recorded with an integration time of 100 ns using an iCCD camera from *Andor*. For the detection of the decay traces at single wavelengths a photomultiplier tube was employed. All transient absorption experiments were performed at 293 K. ^**1**^H NMR spectra were recorded on a Bruker Avance II 400 spectrometer at 400.13 MHz. All resonances are reported in ppm versus the solvent signal as an internal standard (δ = 1.94, CD_3_CN) (Fulmer, et al., 2010).

General Procedure for the light-driven radical cation [4+2] cycloaddition. A solution of *trans*-anethole ***t*An** (100 mM), 2,3-dimethyl-1,3-butadiene **DMB** (500 mM) and the photocatalyst (0.2–5 mM) in acetonitrile with a total volume of 2 ml was prepared in a 4 ml vial. The vial was capped with a septum, pierced with a needle to allow air contact, equipped with a stir bar and placed in an Aldrich® Micro Photochemical Reactor (16 vial places) equipped with blue LEDs (for the LED emission spectrum in combination with the UV/Vis absorption spectra of the photocatalysts see Supplementary Figures S1–S4). The vials were irradiated at 294 K. The progress of the reaction was monitored by ^1^H NMR spectroscopy. To this end an aliquot of the reaction mixture was taken and added to a solution of an internal standard (naphthalene) in CD_3_CN. The yield was determined via integration of the product and starting material resonances versus the internal standard.

Isolation of the product DAP. In order to isolate the **DAP** product, water (2 ml) was added to the reaction mixture and the solution was extracted with diethylether (2 × 2 ml). The organic phase was dried over anhydrous sodium sulfate and the solvent was removed under reduced pressure. The crude product was purified by filtration through a silica-filled glass pipette using cyclohexane. The solvent was removed under reduced pressure to give the product as a colorless oil.

## Results and Discussion

**FIGURE 1:**
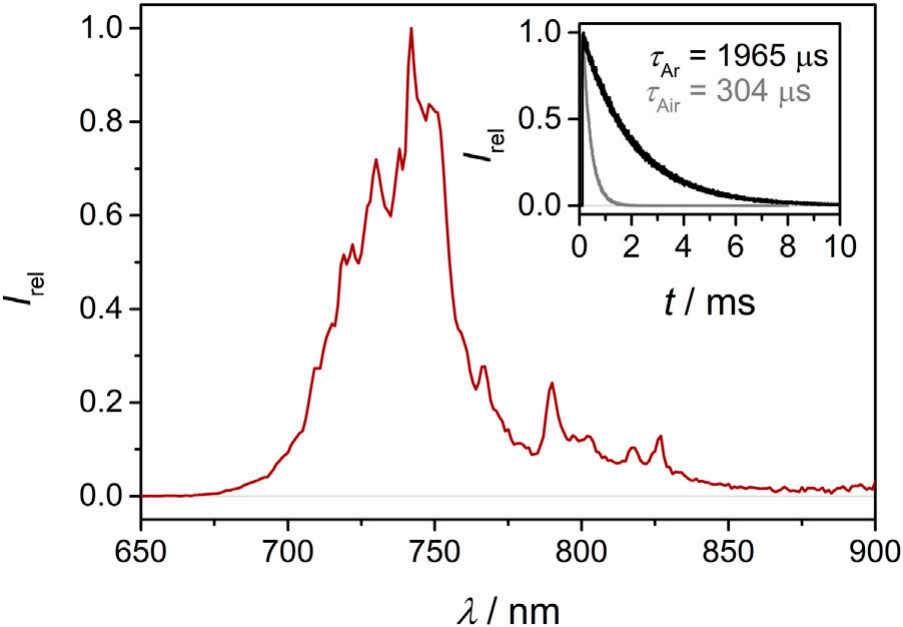
Emission spectrum of **[Cr(tpe)**_**2**_ **][BF**_**4**_ **]**_**3**_ (1 mM) in acetonitrile after excitation at 355 nm. Inset: emission decay curves under deaerated (black) and aerated (gray) conditions.

The photocatalysts **[Cr**(**ddpd)**_**2**_ **]** ^**3+**^, **[Cr**(**tpe)**_**2**_ **]** ^**3+**^, **[Cr**(**dmcbpy)**_**3**_ **]** ^**3+**^ and **[Ru**(**bpz)**_**3**_ **]** ^**2+**^ (**PC** ^**n+**^) employed in this study are depicted in Scheme 1C (Otto, et al., 2015; Treiling, et al., 2019; Crutchley and Lever, 1980). All complexes absorb blue light with absorption maxima at 436, 431, 448 and 440 nm in CH_3_CN, albeit with very different extinction coefficients of *ε*_max_ = 3770, 30, 714 and 14,100 M^−1^ cm^−1^, respectively. Excitation with blue light leads to phosphorescence emission maxima at 776, 748, 733 and 607 nm, respectively. Under inert atmosphere, the corresponding excited state lifetimes amount to 1136, 1965, 25 and 0.762 µs (Figure 1; Supplementary Figures S5–S7) and the photoluminescence quantum yields to 12.1, 4.0, 0.14 and 6.5%, respectively.

Thermodynamically, all chromium(III) complexes **[Cr**(**ddpd)**_**2**_ **]** ^**3+**^, **[Cr**(**tpe)**_**2**_ **]** ^**3+**^ and **[Cr**(**dmcbpy)**_**3**_ **]** ^**3+**^ as well as **[Ru**(**bpz)**_**3**_ **]** ^**2+**^ can sensitize triplet oxygen (^3^O_2_) to give singlet oxygen (^1^O_2_) due to their excited state energies (1.6–2.16 eV; Otto, et al., 2015; Treiling, et al., 2019; Stevenson, et al., 2015; Vleck, et al., 1995) exceeding the energy of ^1^O_2_ (0.97 eV). In aerated CH_3_CN solution, the luminescence lifetimes decrease to 48, 304, 19 and 0.561 µs for **[Cr**(**ddpd)**_**2**_ **]** ^**3+**^, **[Cr**(**tpe)**_**2**_ **]** ^**3+**^, **[Cr**(**dmcbpy)**_**3**_ **]** ^**3+**^ and **[Ru**(**bpz)**_**3**_ **]** ^**2+**^, respectively (Figure 1; Supplementary Figures S5–S7). **[Cr**(**ddpd)**_**2**_ **]** ^**3+**^ had been employed for ^1^O_2_ generation for the α-cyanation of aliphatic amines and in biological environments (Otto, et al., 2015; Otto, et al., 2017; Basu, et al., 2019). The spin-flip phosphorescence of **[Cr(tpe)_2_]^3+^** is likewise quenched by O_2_ (Treiling, et al., 2019). Stern-Volmer studies of **[Cr**(**ddpd)**_**2**_ **]** ^**3+**^, **[Cr**(**tpe)**_**2**_ **]** ^**3+**^, **[Cr**(**dmcbpy)**_**3**_ **]** ^**3+**^ and **[Ru**(**bpz)**_**3**_ **]** ^**2+**^ with O_2_ in CH_3_CN yield K_SV_ = 130 × 10^−3^, 34 × 10^−3^, 1.8 × 10^−3^ and 2.3 × 10^−3^ hPa^−1^ and k_q_ = 115, 17.3, 73.3 and 3010 hPa^−1^ s^−1^, respectively (Supplementary Figures S8–S11). The concentration of ^3^O_2_ in air-saturated CH_3_CN solution has been reported as 1.9 mM (Montalti, et al., 2006). The formation of ^1^O_2_ was confirmed by its characteristic emission band at 1275 nm (Supplementary Figure S12). Its experimental formation and decay was excellently reproduced by a kinetic model shown in the Supplementary Material (Supplementary Figure S12). As ^1^O_2_ is formed and sufficiently long-lived, it might play a role in the catalytic cycle as proposed by Rappé and Shores (Scheme 1A), although its concentration would be quite low (Higgins, et al., 2016). Even in the presence of oxygen, the excited state lifetimes of **[Cr**(**ddpd)**_**2**_ **]** ^**3+**^, **[Cr**(**tpe)**_**2**_ **]** ^**3+**^, **[Cr**(**dmcbpy)**_**3**_ **]** ^**3+**^ and **[Ru**(**bpz)**_**3**_ **]** ^**2+**^ remain high (Figure 1; Supplementary Figures S5–S7) enabling efficient quenching with other substrates in parallel processes such as photoinduced redox processes.

Indeed, the chromium(III) complexes **[Cr**(**ddpd)**_**2**_ **]** ^**3+**^, **[Cr**(**tpe)**_**2**_ **]** ^**3+**^ and **[Cr**(**dmcbpy)**_**3**_ **]** ^**3+**^ can be reduced to the corresponding dications at –1.11, –0.88 and –0.26 V vs. ferrocene, respectively (Otto, et al., 2015; Treiling, et al., 2019; McDaniel, et al., 2010). The ruthenium(II) complex **[Ru**(**bpz)**_**3**_ **]** ^**2+**^ can be reduced at –1.11 V whereby the additional electron occupies a ligand-centered π* orbital (Campagna, et al., 2007; Balzani, et al., 1978). Reduced chromium(III) complexes are either genuine chromium(II) complexes for electron-rich ddpd ligands (Becker, et al., 2020) or ligand centered radicals for electron-deficient tpe, bpmp or bpy-type ligands (bpmp = 2,6-bis(2-pyridylmethyl)pyridine; Treiling, et al., 2019; Reichenauer, et al., 2020; Scarborough, et al., 2011). While metal-centered reduction to Cr^II^ can labilize ligands, ligand-centered radicals appear comparably stable similar to ligand-reduced ruthenium(II) complexes such as **[Ru**(**bpz)**_**3**_ **]** ^**+**^ (Campagna, et al., 2007; Balzani, et al., 1978; Lin, et al., 2011).

**FIGURE 2:**
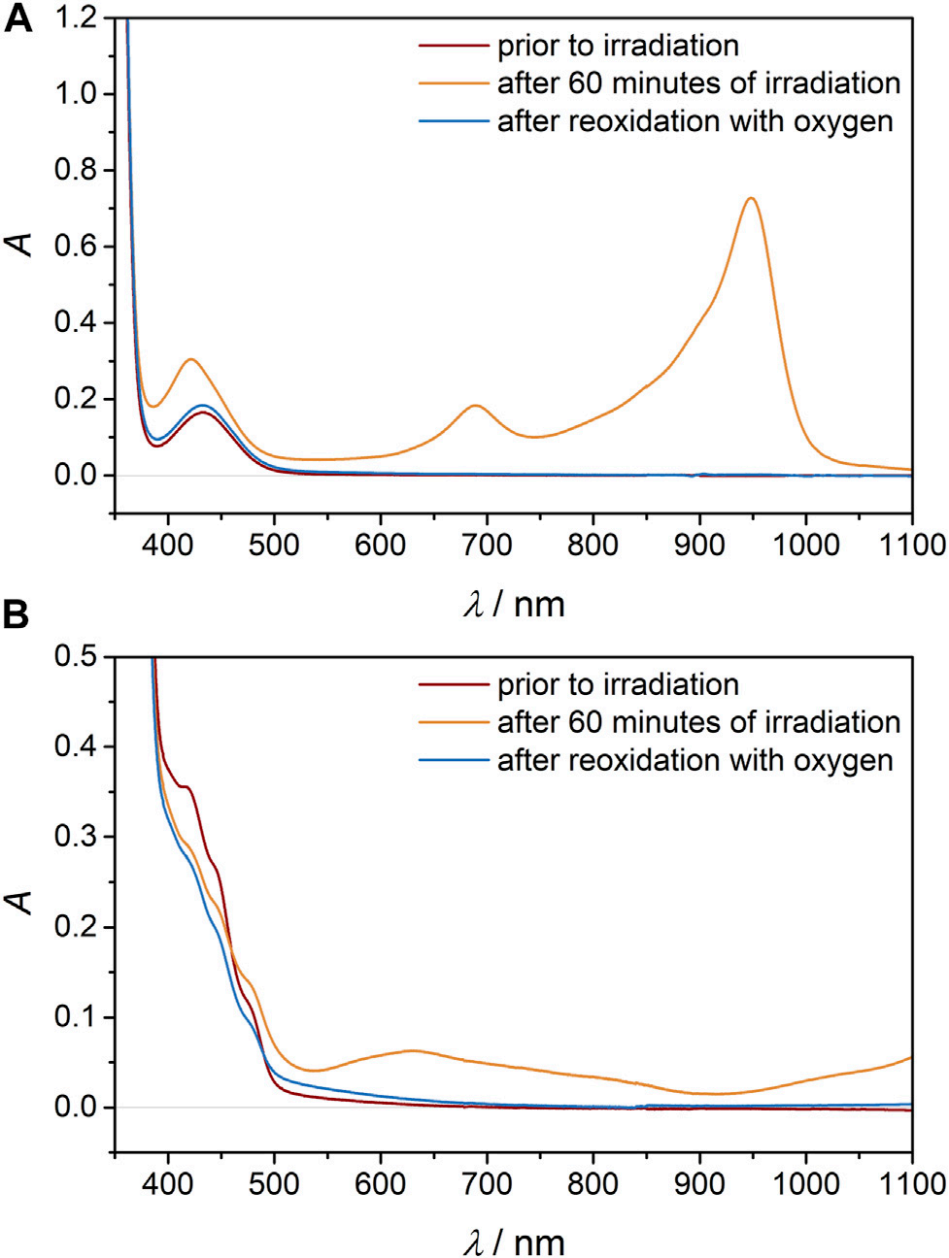
UV/vis/NIR absorption spectra of **(A) [Cr(tpe)**_**2**_ **]** ^**3+**^ and **(B) [Cr(dmcbpy)**_**3**_ **]** ^**3+**^ in CH_3_CN prior to irradiation (red), after 60 min of LED irradiation (λ_exc_ = 460 nm) under argon in the presence of ***t*An** (orange) and after re-oxidation with air in the dark (blue).

[Cr(bpy)_3_]^3+^, [Cr(tpy)_2_]^3+^ and related derivatives photooxidize DNA via initial guanine base oxidation (tpy = 2,2’; 6′,2″-terpyridine) while *[Cr(**ddpd)**_**2**_ **]** ^**3+**^ does not oxidize guanine bases due to its low excited state redox potential E_**½**_ ([*Cr(ddpd)_**2**_ **]** ^**3+**^/**[Cr**(**ddpd)**_**2**_ **]** ^**2+**^) = 0.49 V(Baptista, et al., 2021; Watson, et al., 1999; Vaidyanathan, et al., 2004; Otto, et al., 2015). We will exploit the selectivity of *[Cr(**ddpd)**_**2**_ **]** ^**3+**^ for energy transfer over electron transfer later in mechanistic studies (Otto, et al., 2015). On the other hand, the excited state potentials of **[Cr**(**tpe)**_**2**_ **]** ^**3+**^ and **[Cr**(**dmcbpy)**_**3**_ **]** ^**3+**^ of 0.87 and 1.44 V approach or even exceed that of **[Ru**(**bpz)**_**3**_ **]** ^**2+**^ with 0.99 V. Hence, **[Cr**(**tpe)**_**2**_ **]** ^**3+**^, **[Cr**(**dmcbpy)**_**3**_ **]** ^**3+**^ and **[Ru**(**bpz)**_**3**_ **]** ^**2+**^ are strong photooxidants capable of oxidizing ***t*An** to ***t*An** ^**•+**^ (E_½_ = 0.78 V; Higgins, et al., 2016), while **[Cr**(**ddpd)**_**2**_ **]** ^**3+**^ is thermodynamically incompetent for this photoredox reaction. Consequently, ***t*An** reductively quenches the emission of **[Cr**(**tpe)**_**2**_ **]** ^**3+**^, **[Cr**(**dmcbpy)**_**3**_ **]** ^**3+**^ and **[Ru**(**bpz)**_**3**_ **]** ^**2+**^ but not that of **[Cr**(**ddpd)**_**2**_ **]** ^**3+**^. Stern-Volmer and quenching rate constants of **[Cr**(**tpe)**_**2**_ **]** ^**3+**^, **[Cr**(**dmcbpy)**_**3**_ **]** ^**3+**^ and **[Ru**(**bpz)**_**3**_ **]** ^**2+**^ with ***t*An** were determined as K_SV_ = 4500, 75,000 and 740 M^−1^ and k_q_ = 2.3×10^6^, 3000×10^6^ and 970×10^6^ M^−1^ s^−1^, respectively (Supplementary Figures S13–S15). The different rates reflect the different driving forces for the electron transfer from ***t*An** to the excited photocatalyst *PC ^**n+**^ (Marcus, 1993). **[Cr**(**tpe)**_**2**_ **]** ^**2+**^ forms by reduction of **[Cr**(**tpe)**_**2**_ **]** ^**3+**^ with ***t*An** under light irradiation in the photo reactor as confirmed by UV/Vis spectroscopy and comparison with an authentic spectrum (Figure 2A) (Treiling, et al., 2019) Similarly **[Cr**(**dmcbpy)**_**3**_ **]** ^**3+**^ is reduced to **[Cr**(**dmcbpy)**_**3**_ **]** ^**2+**^ (Figure 2B) by reference to spectra of [CrL_3_]^2+^ complexes with bpy-type ligands (Scarborough, et al., 2011; Higgins, et al., 2016).

Interestingly, addition of O_2_ to the **[Cr**(**tpe)**_**2**_ **]** ^**2+**^ solution in the dark restores the UV/vis/NIR absorption spectrum of **[Cr**(**tpe)**_**2**_ **]** ^**3+**^ quantitatively demonstrating that ^3^O_2_ can oxidize **[Cr**(**tpe)**_**2**_ **]** ^**2+**^ to **[Cr**(**tpe)**_**2**_ **]** ^**3+**^ (Figure 2A). Similarly, the UV/vis/NIR absorption spectrum of **[Cr**(**dmcbpy)**_**3**_ **]** ^**3+**^ is recovered by addition of O_2_ to the **[Cr**(**dmcbpy)**_**3**_ **]** ^**3+**^ solution in the dark at least to a large extent (Figure 2B). This points to some instability either of **[Cr**(**dmcbpy)**_**3**_ **]** ^**2+**^ or the photoexcited complex *[Cr(**dmcbpy)**_**3**_ **]** ^**3+**^. In fact, **[Cr**(**tpe)**_**2**_ **]** ^**3+**^ is photostable in CH_3_CN over several days of irradiation while **[Cr**(**dmcbpy)**_**3**_ **]** ^**3+**^ and **[Ru**(**bpz)**_**3**_ **]** ^**2+**^ partially decompose over time irrespective of the presence or absence of O_2_ (Supplementary Figures S16–S21). The recovery of **[Cr**(**tpe)**_**2**_ **]** ^**3+**^ and **[Cr**(**dmcbpy)**_**3**_ **]** ^**3+**^ by oxygen in the absence of light clearly demonstrates that in contradiction to the previously proposed mechanism no ^1^O_2_ is needed to reoxidize the catalyst. This surprising finding motivated us to perform LFP investigations on **[Cr**(**tpe)**_**2**_ **]** ^**3+**^ to unravel the mechanism and importantly to elucidate the role of oxygen in this photoredox catalytic cycle.

**FIGURE 3:**
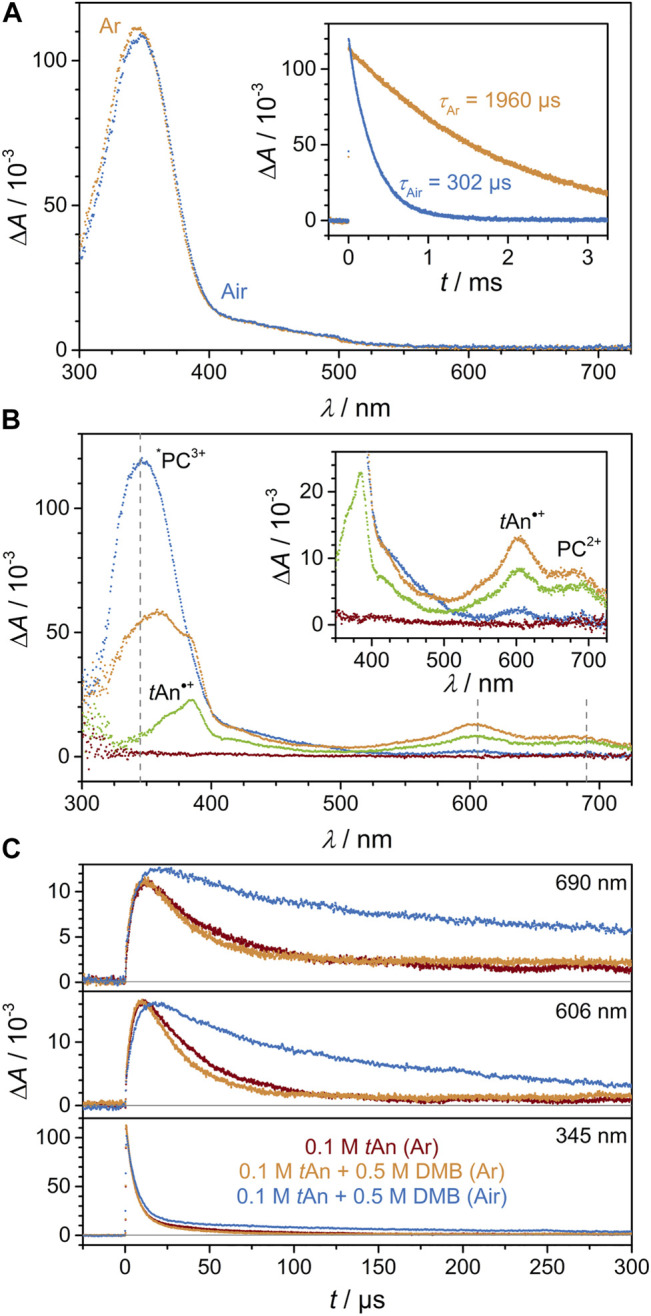
LFP investigations on pure and quenched solutions of **[Cr(tpe)**_**2**_ **][BF**_**4**_ **]**_**3**_ (0.35 mM) in acetonitrile illustrating the evolution and the decay of the key intermediates ^*^ **PC** ^**3+**^, **PC** ^**2+**^ and ***t*An**•^**+**^ following laser excitation at 355 nm. **(A)** Transient absorption spectra of an air-saturated (blue) and a deaerated (orange) **[Cr(tpe)**_**2**_ **][BF**_**4**_ **]**_**3**_ solution at a time delay of 50 ns. Inset, corresponding decay traces at 345 nm. **(B)** Transient absorption spectra monitored 50 ns (blue), 5 µs (orange), 50 µs (green) and 500 µs (red) after laser excitation of a deaerated **[Cr(tpe)**_**2**_ **][BF**_**4**_ **]**_**3**_ solution in acetonitrile quenched by 0.1 M ***t*An**. **(C)** Decay traces at 345, 606 and 690 nm upon excitation of **[Cr(tpe)**_**2**_ **][BF**_**4**_ **]**_**3**_ solutions with 0.1 M ***t*An** (deaerated, red) and 0.1 M ***t*An** and 0.5 M **DMB**, respectively (deaerated, orange; air-saturated, blue).

A solution of **[Cr**(**tpe)**_**2**_ **]** ^**3+**^ in deaerated acetonitrile was excited at 355 nm. Figure 3A illustrates the resulting transient absorption spectrum, which is dominated by a strong positive absorption band centered at 345 nm. According to time-dependent Density Functional Theory calculations, this absorption band covers spin-allowed transitions with doublet ligand-to-metal and intraligand character calculated at 345 and 373 nm (Supplementary Tables S1, S2). Analogous quartet transitions are calculated at 325 nm for the quartet ground state. The assignments fit to the observed experimental band maxima of 329 nm (Supplementary Figure S3) and 345 nm for ^**4**^ **[Cr**(**tpe)**_**2**_ **]** ^**3+**^ and ^**2**^ **[Cr**(**tpe)**_**2**_ **]** ^**3+**^, respectively. The decay trace observed at 345 nm for ^**2**^ **[Cr**(**tpe)**_**2**_ **]** ^**3+**^ shows a first order decay with a time constant of 1960 µs. In air-saturated solution the absorption spectrum remains unaltered, but the lifetime substantially decreases to 302 μs, compared to the deaerated solution. Both lifetimes excellently agree with the emission lifetimes determined via time-resolved emission (Figures 1, 3A).

Excitation of a deaerated solution of **[Cr**(**tpe)**_**2**_ **]** ^**3+**^ in the presence of 0.1 M ***t*An** initially yields the same absorption spectrum as the photocatalyst alone showing that no observable intermediates are formed due to direct excitation of ***t*An** (Figure 3B). The *PC ^**3+**^ absorption (345 nm) rapidly decays with a lifetime of *ca*. 5 μs (Figure 3C), which agrees with the time constant found for the emission quenching (Supplementary Figure S14, τ_0_ = 1965 µs, K_SV_ = 4520 M^−1^, 0.1 M ***t*An**). Two new bands appear at 385 and 606 nm (Figure 3B) that coincide with the absorption maxima reported for ***t*An** ^**•+**^ by Johnston and Schepp (Johnston and Schepp, 1993) confirming the formation of the radical cation by reductive quenching of *PC ^**3+**^. An additional weak band was observed at 690 nm that can be assigned to **PC** ^**2+**^ by comparison with an authentic spectrum (Figure 2A; Treiling, et al., 2019), albeit ***t*An** ^**•+**^ also significantly contributes to the absorption at this wavelength. Figure 3C (red curves) displays the decay traces at the absorption maxima 345, 606 and 690 nm, corresponding to *PC ^**3+**^, ***t*An** ^**•+**^ and **PC** ^**2+**^, respectively. Interestingly, the kinetic traces at 606 and 690 nm show a first-order decay with a lifetime of *ca*. 45 μs (first measurement with a fresh sample) after an initial rise period of approximately 20 µs. This finding is unexpected for two reasons: the radical cation ***t*An** ^**•+**^ is much more long-lived than described by Johnston and Schepp (Johnston and Schepp, 1993) and the unproductive non-geminate back-electron transfer (back-ET) from **PC** ^**2+**^ to ***t*An** ^**•+**^ should be the dominant deactivation process in the absence of oxygen or **DMB**, which would yield a second-order decay. This deviation from the expected second-order rate law can be rationalized by a build-up of **PC** ^**2+**^ during the LFP experiment, which can lead to pseudo-first order kinetics. Although **PC** ^**2+**^ and ***t*An** ^**•+**^ are initially formed in equal amounts, **PC** ^**2+**^ can accumulate by removing a fraction of ***t*An** ^**•+**^ via a side reaction. In fact, ***t*An** ^**•+**^ is known to undergo a variety of reactions besides back electron transfer, e.g. addition to ***t*An**, nucleophilic addition and dimerization (Johnston and Schepp, 1993; Johnston and Schepp 1995). In principle, the addition of ***t*An** ^**•+**^ to ***t*An** alone could also explain the observed pseudo first-order rate law. However, only traces of side products were observed upon irradiation with a LED, which reveals that only a small fraction of ***t*An** ^**•+**^ follows this reaction path. Yet this small amount of removed ***t*An** ^**•+**^ would suffice to explain the accumulation of **PC** ^**2+**^ as the ***t*An** concentration exceeds the concentration of **[Cr**(**tpe)**_**2**_ **]** ^**3+**^ by approximately two orders of magnitude. The substantial residual signal at 690 nm provides further evidence for this incomplete reoxidation of **PC** ^**2+**^ to **PC** ^**3+**^. In order to test whether **PC** ^**2+**^ accumulates during the LFP experiments, we performed repeated measurements of the decay trace at 606 nm (eight iterations) with the same sample. In this series of experiments the lifetime successively decreases to a limiting value of 23 µs (Supplementary Figure S22). Moreover, the static UV/vis/NIR spectrum recorded after the LFP measurement clearly shows the characteristic spectral signature of **PC** ^**2+**^ (Supplementary Figure S23).

In order to observe the [4+2] cycloaddition step of ***t*An** ^**•+**^ to **DMB** to give **DAP** ^**•+**^ (Scheme 1B), LFP measurements were conducted with additional 0.5 M **DMB**. However, the obtained transient absorption spectra in the presence and absence of **DMB** are basically identical (Supplementary Figure S24). Consequently, the formation of **DAP** ^**•+**^ cannot be monitored under these conditions. Figure 3C (orange curve) illustrates the corresponding decay traces. Despite of the large excess of **DMB**, the decay rate observed at 606 nm (***t*An** ^**•+**^) and 690 nm (**PC** ^**2+**^) is only slightly increased. The very slow kinetics might be explained by the preferred *s*-*trans* conformation (by 8.3 kJ mol^−1^; Squillacote and Liang, 2005) of **DMB** and the steric demands of the diene **DMB** and the dienophile ***t*An** ^**•+**^ posing a barrier for the addition/cyclization step to **DAP** ^**•+**^. This argument also rationalizes the missing accumulation of **DAP** ^**•+**^. Hence, the slow [4+2] cycloaddition of **DMB** with ***t*An** ^**•+**^ is probably the bottleneck of the reaction sequence. In agreement with this interpretation, the first C–C bond formation of the asynchronous radical cation [4+2] cycloaddition has been identified computationally to be the most demanding step (Young, et al., 2017).

Finally, LFP measurements were performed on an air-saturated solution of **[Cr**(**tpe)**_**2**_ **]** ^**3+**^, 0.1 M ***t*An** and 0.5 M **DMB**. The shape of the absorption spectra remains unaffected by the presence of oxygen (Supplementary Figure S25). Surprisingly, the presence of oxygen strongly *prolongs* the lifetime of ***t*An** ^**•+**^ (Figure 3C, blue curves). This contrasts the reported reaction of ***t*An** ^**•+**^ (prepared by direct photoionization at 308 or 266 nm) with O_2_ (Johnston and Schepp, 1993), which *shortens* the lifetime significantly (*k*_O2_ = 2.3×10^7^ M^−1^s^−1^). The decay of ***t*An** ^**•+**^ (prepared by photoinduced electron transfer to **PC** ^**3+**^) in the presence of O_2_ can be reasonably well described with second order kinetics (Supplementary Figure S26) in contrast to the first order decay found for the Ar-saturated samples. We surmise that ^3^O_2_ oxidizes **PC** ^**2+**^ recovering the catalyst in its initial form, which prevents the accumulation of **PC** ^**2+**^ during the LFP measurement and also during the continuous irradiation in the laboratory scale experiments. Consequently, the non-geminate back-ET from **PC** ^**2+**^ to ***t*An** ^**•+**^ is slowed down by the removal of **PC** ^**2+**^ with O_2_, which enhances the lifetime of ***t*An** ^**•+**^. Interestingly, the lifetime of ***t*An** ^**•+**^ further increases, when the decay trace at 606 nm is repeatedly recorded with the same sample but stays constant after the sixth iteration (Supplementary Figure S26). This observation might be explained by photochemical self-cleaning, which removes trace impurities in the catalyst solution.

In summary, the collected data support the simplified productive mechanistic picture outlined in Scheme 1B. This scheme includes a non-productive back-ET pathway, but lacks a second photocycle proposed in Scheme 1A involving ^1^O_2_. Excitation of the Cr^III^ photocatalyst initially yields a quartet excited state (^4^T_2_ or ^4^CT states), which evolve to the productive doublet states via ISC. While **[Cr**(**dmcbpy)**_**3**_ **]** ^**3+**^ suffers from occasional photodissociation, **[Cr**(**tpe)**_**2**_ **]** ^**3+**^ is stable towards photodissociation and photosubstitution. Both complexes can form ^1^O_2_ from ^3^O_2_ in their long-lived excited doublet states but with comparably low efficiency. In fact, our studies clearly demonstrate that ^1^O_2_ is unnecessary to regenerate **PC** ^**3+**^ from **PC** ^**2+**^ as ^3^O_2_ is already competent in spite of its formal reduction potential being very negative (Singh, et al., 2006). Hydrogen-bond forming water has been reported to shift the O_2_^•–^/^3^O_2_ potential to less negative values and protonation of O_2_^•–^ to HO_2_^•^ might even drive the redox reaction due to O_2_^•–^ consumption, which might explain the **PC** ^**n+**^ regeneration competency of the O_2_^•–^/^3^O_2_ pair in non-dried CH_3_CN (Singh, et al., 2006). Consequently, ^1^O_2_ as proposed in Scheme 1A is not mandatory for the productive catalytic cycle (Scheme 1B). Reductive quenching of the long-lived excited doublet states of **PC** ^**3+**^ by the styrene ***t*An** is highly efficient forming the reduced photocatalyst **PC** ^**2+**^ and the radical cation ***t*An** ^**•+**^. In the absence of O_2_, **PC** ^**2+**^ can recombine with ***t*An** ^**•+**^ in a non-productive back-ET due to the microsecond lifetime of ***t*An** ^**•+**^ in the absence of substrates. Even in the presence of excess **DMB**, the lifetime of ***t*An** ^**•+**^ exceeds microseconds suggesting that the addition of ***t*An** ^**•+**^ to **DMB** is the rate-determining step for the catalytic cycle. Shores and others also noticed that the cycloaddition step is slow with terminally substituted dienes but is positively influenced by the presence of O_2_ (Higgins, et al., 2016). In the presence of O_2_, **PC** ^**2+**^ is re-oxidized sufficiently fast by ^3^O_2_ to give **PC** ^**3+**^ and O_2_^•–^. The removal of **PC** ^**2+**^ prolongs the lifetime of ***t*An** ^**•+**^ increasing the probability for the radical addition step. Both **PC** ^**2+**^ and O_2_^•–^ are thermodynamically capable to reduce the product cyclohexene radical cation **DAP** ^**•+**^ to the final product. The radical cation **DAP** ^**•+**^ is not observed by LFP neither in the presence nor absence of O_2_ suggesting that its reduction by **PC** ^**2+**^ is faster than its formation (Scheme 1B). Consequently, the main task of O_2_ is to rapidly remove **PC** ^**2+**^ to prevent non-geminate back-ET from **PC** ^**2+**^ to ***t*An** ^**•+**^ and thus to enable the slow addition reaction of ***t*An** ^**•+**^ to **DMB**, while O_2_^•–^ is not necessarily required to reduce **DAP** ^**•+**^. Furthermore, rapid regeneration of **PC** ^**3+**^ prevents accumulation of the highly absorptive one-electron reduced photocatalyst **PC** ^**2+**^ **[Cr**(**tpe)**_**2**_ **]** ^**2+**^ (Figure 2A) which would absorb a significant amount of the incoming photons.

**FIGURE 4:**
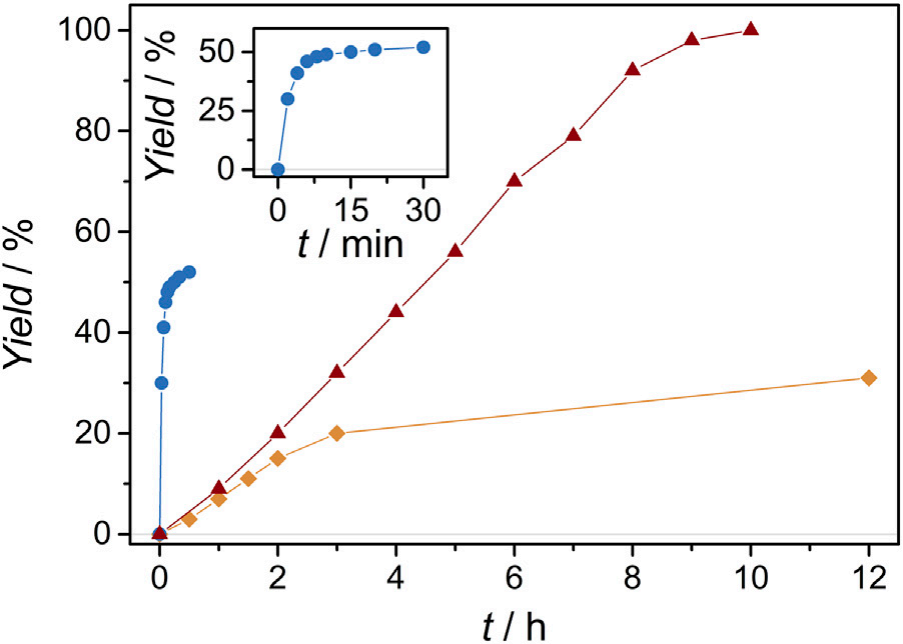
Conversion vs. time plots of the photoredox catalyzed radical cation Diels-Alder cycloaddition of ***t*An** and **DMB** using **[Cr(tpe)**_**2**_ **]** ^**3+**^ (red triangles), **[Cr(dmcbpy)**_**3**_ **]** ^**3+**^ (orange diamonds) and **[Ru(bpz)**_**3**_ **]** ^**2+**^ (blue circles) photocatalysts (0.2 mM) in CH_3_CN with 460 nm light excitation. The inset shows a zoom of the conversion vs. time plot of the rapid but incomplete reaction with **[Ru(bpz)**_**3**_ **]** ^**2+**^ as photocatalyst.

The competent photocatalysts **[Cr**(**tpe)**_**2**_ **]** ^**3+**^, **[Cr**(**dmcbpy)**_**3**_ **]** ^**3+**^ and **[Ru**(**bpz)**_**3**_ **]** ^**2+**^ were employed in catalytic experiments with ***t*An** and **DMB** substrates in air-saturated CH_3_CN under visible light excitation (460 nm). The overlap of the absorption spectra of **PC** ^**n+**^ and the emission of the LEDs employed reveal that photon absorption in this spectral region increases in the series **[Cr**(**tpe)**_**2**_ **]** ^**3+**^, **[Cr**(**dmcbpy)**_**3**_ **]** ^**3+**^ and **[Ru**(**bpz)**_**3**_ **]** ^**2+**^ due to the weak Laporte-forbidden absorption bands of the Cr^III^ photocatalysts in the visible spectral region. This is particularly concerning for the centrosymmetric complex **[Cr**(**tpe)**_**2**_ **]** ^**3+**^ with an extinction coefficient at its absorption maximum of only ε_431_ = 30 M^−1^ cm^−1^ as compared to the much higher extinction coefficients of **[Cr**(**dmcbpy)**_**3**_ **]** ^**3+**^ (ε_448_ = 714 M^−1^ cm^−1^) and **[Ru**(**bpz)**_**3**_ **]** ^**2+**^ (ε_440_ = 14,100 M^−1^ cm^−1^). Consequently, we employed a loading of 5 mM **[Cr**(**tpe)**_**2**_ **]** ^**3+**^ in initial catalytic tests. According to ^1^H NMR spectroscopic reaction monitoring (Supplementary Figures S27–S31), full conversion to the Diels-Alder product **DAP** is achieved with **[Cr**(**tpe)**_**2**_ **]** ^**3+**^ within ca. 80 min (Supplementary Figure S32). In the absence of O_2_ only traces of **DAP** were observed after 4 h irradiation (Supplementary Figure S33). Introduction of O_2_ to this pretreated reaction mixture initiates the catalytic turnover and the reaction proceeds quantitatively. This confirms that the unproductive back-ET efficiently prevents the formation of **DAP** ^**•+**^. In the absence of light (24 h) or **[Cr**(**tpe)**_**2**_ **]** ^**3+**^ (24 h) **DAP** formation is not observed (Supplementary Figures S34, S35). In the latter case, traces of 4-anisaldehyd and other byproducts are formed (Supplementary Figure S35). Reducing the catalyst loading to 1 and 0.2 mM prolongs the reaction time to reach full conversion to ca. 5–6 and 9–10 h, respectively (Figure 4; Supplementary Figure S36). Notably, full conversion is achieved with **[Cr**(**tpe)**_**2**_ **]** ^**3+**^ in all cases. The solutions remain only weakly colored during the photolysis experiments suggesting a high stability of **[Cr**(**tpe)**_**2**_ **]** ^**3+**^, *[Cr(**tpe)**_**2**_ **]** ^**3+**^ and **[Cr**(**tpe)**_**2**_ **]** ^**2+**^. In fact, addition of new ***t*An** and **DMB** substrates to the catalysis solution after full conversion, resumes the catalytic activity without noticeable degradation as demonstrated two times (Supplementary Figure S37). The high photo and redoxstability of **[Cr**(**tpe)**_**2**_ **]** ^**3+**^ (Supplementary Figure S38) and the resulting recyclability (Supplementary Figure S37) are unique to **[Cr**(**tpe)**_**2**_ **]** ^**3+**^ (Figure 4).

**[Ru**(**bpz)**_**3**_ **]** ^**2+**^ reacts initially much faster than the Cr^III^ photocatalysts **[Cr**(**dmcbpy)**_**3**_ **]** ^**3+**^ and **[Cr**(**tpe)**_**2**_ **]** ^**3+**^ with initial rates being 300 × 10^−4^, 2.5 × 10^−4^ and 4.3 × 10^−4^ mM min^−1^ and turnover frequencies being TOF = 75, 0.6 and 1.1 min^−1^, respectively (loading 0.2 mM; Figure 4). Importantly, **[Cr**(**dmcbpy)**_**3**_ **]** ^**3+**^ and **[Ru**(**bpz)**_**3**_ **]** ^**2+**^ do not achieve full conversion under these conditions (loading 0.2 mM) but the catalytic activity levels off at ca. 30 and 50% conversion after 720 and 6 min, respectively. Photodecomposition of the **[Cr**(**dmcbpy)**_**3**_ **]** ^**3+**^ and **[Ru**(**bpz)**_**3**_ **]** ^**2+**^ with ligand loss is likely responsible for this behavior. Indeed, the UV/Vis spectra of the solutions after the catalysis changed for **[Cr**(**dmcbpy)**_**3**_ **]** ^**3+**^ and **[Ru**(**bpz)**_**3**_ **]** ^**2+**^, but not for **[Cr**(**tpe)**_**2**_ **]** ^**3+**^ (Supplementary Figures S38–S40). Although conditions for chromium(III) and ruthenium(II) complexes with bidentate ligands can be optimized to achieve full conversion, re-using these photocatalysts has not yet been reported (Lin, et al., 2011; Stevenson, et al., 2015; Farney, et al., 2019).

Only traces of byproducts form during the reaction with **[Cr**(**tpe)**_**2**_ **]** ^**3+**^ as photocatalyst. Discernible ^1^H NMR resonances at δ = 9.84, 7.82, 6.87 and 3.86 ppm suggest the formation of 4-anisaldehyde arising from the reaction of ***t*An** ^**•+**^ with oxygen (<< 1%; Supplementary Figure S41) (Lewis and Kijima, 1988) A further byproduct with ^1^H NMR resonances around 8.8 ppm also likely arises from a ***t*An** ^**•+**^ derived species but could not be definitely assigned, yet various dimers and other oxygenated products are conceivable (Nozaki, et al., 1968). Side reactions of ***t*An** with ^1^O_2_ are excluded as a photocatalytic control reaction using **[Cr**(**ddpd)**_**2**_ **]** ^**3+**^ as **PC** ^**3+**^ which is merely capable to form ^1^O_2_ but not ***t*An** ^**•+**^ does not yield any products.

At first glance, molecular rubies seem to be rather unattractive in the context of photochemical applications, such as photoredox catalysis, due to their low absorbance in the visible part of the spectrum, which appears to imply an inefficient use of the incident photons. This seems especially true for **[Cr**(**tpe)**_**2**_ **]** ^**3+**^, which has an extinction coefficient as low as 30 M^−1^ cm^−1^ at the maximum of the band in the visible region. However, for one-photon processes and a reasonable reactor design, this turns out to be not the case, especially when the photocatalyst is as stable (reusable) and easy to access as **[Cr**(**tpe)**_**2**_ **]** ^**3+**^. Laboratory scale applications typically go hand in hand with a moderate upscaling compared to the mechanistic studies that are typically conducted in a cuvette or vial. On a preparative laboratory scale it is convenient to conduct the reaction in a round bottom flask or a larger cuvette, such an optical path of 10 cm or more is likely achieved. In this case, **[Cr**(**tpe)**_**2**_ **]** ^**3+**^ photocatalyst loadings of 1 and 5 mM give absorbances of 0.3 and 1.5, respectively. According to the Lambert-Beer law 50 and 97% of the incident photons are absorbed, which is a fairly efficient use of light. In fact, the low extinction coefficient of **[Cr**(**tpe)**_**2**_ **]** ^**3+**^ and other molecular rubies might be even beneficial in some reactions as byproduct formation can be decreased by mitigating high local concentrations of radical intermediates as opposed to reactions with strongly absorbing catalysts.

## Conclusion

The chromium(III)-based photocatalysts **PC** ^**3+**^ **[Cr**(**dmcbpy)**_**3**_ **]** ^**3+**^ and **[Cr**(**tpe)**_**2**_ **]** ^**3+**^ catalyze the visible light-driven radical cation [4+2] cycloaddition of *trans*-anethole ***t*An** and 2,3-dimethyl-1,3-butadiene **DMB** using visible light similar to the highly oxidative ruthenium(II) photocatalyst **[Ru**(**bpz)**_**3**_ **]** ^**2+**^. The chromium(III) photocatalysts **PC** ^**3+**^ are quenched reductively by ***t*An** to give **PC** ^**2+**^ and ***t*An** ^**•+**^. The reaction of ***t*An** ^**•+**^ with **DMB** to the **DAP** ^**•+**^ product radical cation is the slowest step of the catalytic cycle. Therefore, oxygen is required for efficient performance to retard unproductive non-geminate back-electron transfer from **PC** ^**2+**^ to the long-lived radical cation ***t*An** ^**•+**^ by rapid oxidative regeneration of the photocatalyst. The concomitantly formed superoxide and the reduced photocatalyst **PC** ^**2+**^ reduce the product radical cation **DAP** ^**•+**^ to the Diels-Alder product **DAP** so that **DAP** ^**•+**^ does not accumulate. In contrast to **[Cr**(**dmcbpy)**_**3**_ **]** ^**3+**^ and **[Ru**(**bpz)**_**3**_ **]** ^**2+**^ the molecular ruby **[Cr**(**tpe)**_**2**_ **]** ^**3+**^ is exceptionally photo and redoxstable and possesses an ultralong excited state lifetime in the millisecond range. Quantitative conversion is achieved contrary to the photolabile photocatalysts **[Cr**(**dmcbpy)**_**3**_ **]** ^**3+**^ and **[Ru**(**bpz)**_**3**_ **]** ^**2+**^. Furthermore, recycling of **[Cr**(**tpe)**_**2**_ **]** ^**3+**^ is successful without significant loss of activity. This mechanistic and exploratory study of the photoredox properties of a molecular ruby paves the way for an efficient and sustainable utilization of photocatalysts based on Earth-abundant chromium ions, in particular photo- and redoxstable molecular rubies, in oxidative photoredox catalysis.

## Data Availability

The original contributions presented in the study are included in the article/Supplementary Material, further inquiries can be directed to the corresponding authors.
