# Supplementary material for: Molecular Rubies in Photoredox Catalysis
Source: Front Chem. 2022 Apr 7;10:887439. doi: 10.3389/fchem.2022.887439 (PMC9021569; doi:10.3389/fchem.2022.887439)
Supplement: Supplementary file 1 [file DataSheet1.PDF]

Supplementary Material

Static unrestricted Kohn-Sham orbitals Density Functional Theory Calculations were performed using the quantum computing suite ORCA 4.0.1 or 4.2.1.[1] Geometry optimization was performed using unrestricted Kohn-Sham orbitals DFT and the B3LYP functional[2] in combination with Ahlrichs' split-valence triple-ζ basis set def2-TZVPP for all atoms.[3,4] Tight convergence criteria were chosen for DFT-UKS calculations (keywords *tightscf* and *tightopt*). All DFT-UKS calculations make use of the resolution of identity (Split-RI-J) approach for the Coulomb term in combination with the chain-of-spheres approximation for the exchange term (*COSX*).[5,6] The zero order relativistic approximation was used to describe relativistic effects in all calculations (keyword *ZORA*).[7,8] Grimme's empirical dispersion correction D3(BJ) was employed (keyword *D3BJ*).[9,10] To account for solvent effects, a conductor-like screening model (keyword *CPCM* acetonitrile) modeling acetonitrile was used in all calculations.[11] A numerical frequency calculation confirmed that the optimized geometry corresponds to a minimum structure. Explicit counter ions and/or solvent molecules were neglected.

Table 1. Cartesian coordinates of the DFT-optimized geometry of $^2[Cr(tpe)_2]^{3+}$.

24	-0.000216963	0.000002117	0.000128061
7	0.062359835	-2.044531336	0.102885816
7	2.044921340	-0.050287185	0.127906904
7	-0.020940073	-0.000768895	2.047243369
6	-0.079570266	0.000681051	4.801326253
6	1.731309206	-1.741336056	1.902580068
6	4.164236534	0.744984198	-0.625736222
6	4.024408325	-0.889061171	1.119616290
6	4.792774220	-0.081350948	0.292805943
6	2.787047451	0.733882059	-0.675459833
6	0.798228954	-0.829842003	2.724118222
6	-0.914398178	0.848723048	4.090411545
6	-0.855388035	0.818215979	2.713956431
6	0.780674028	-0.842079227	4.111192335
6	2.582402438	-2.598976269	2.836705444
6	0.877028209	-2.645427978	0.991680281
6	0.925429936	-4.030273196	1.061321066
6	0.133698219	-4.794718417	0.215625970
6	-0.699835853	-4.161074170	-0.692799913
6	-0.707378217	-2.783152178	-0.717871272
6	2.640738409	-0.863447934	1.020763863
7	-0.062216957	2.044504348	-0.102405852
7	-2.045376961	0.050045880	-0.127178227
7	0.019710265	0.001600761	-2.046906283
6	0.075387120	-0.000693751	-4.801037322
6	-1.732482921	1.742226132	-1.900865005
6	-4.164446088	-0.744039087	0.628414917
6	-4.025406353	0.892164266	-1.114948946
6	-4.793446275	0.084358791	-0.287943862
6	-2.787173395	-0.734372077	0.676239311
6	-0.800239828	0.830443149	-2.723090031
6	0.911262273	-0.848249963	-4.090785144
6	0.853611058	-0.817307911	-2.714282404
6	-0.784235920	0.842139024	-4.110206478
6	-2.583875138	2.600237299	-2.834350076
6	-0.877184317	2.645893124	-0.990543079
6	-0.924883825	4.030770094	-1.060083849
6	-0.132113863	4.794744346	-0.214935923
6	0.701790105	4.160578331	0.692796209
6	0.708505893	2.782667451	0.717770200
6	-2.641602026	0.864674037	-1.018405320

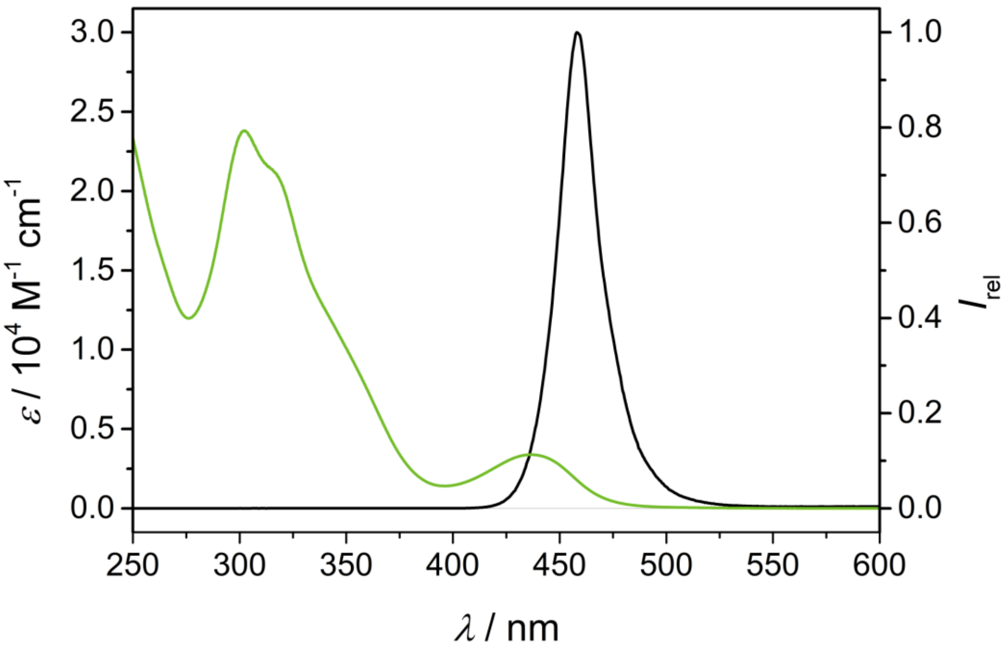

Supplementary Figure 1. UV/Vis spectrum of **[Cr(ddpd)$_2$][BF$_4$]$_3$** (0.1 mM) in acetonitrile (green) and emission spectrum of the Aldrich® Micro Photochemical Reactor, blue LED lights (black).

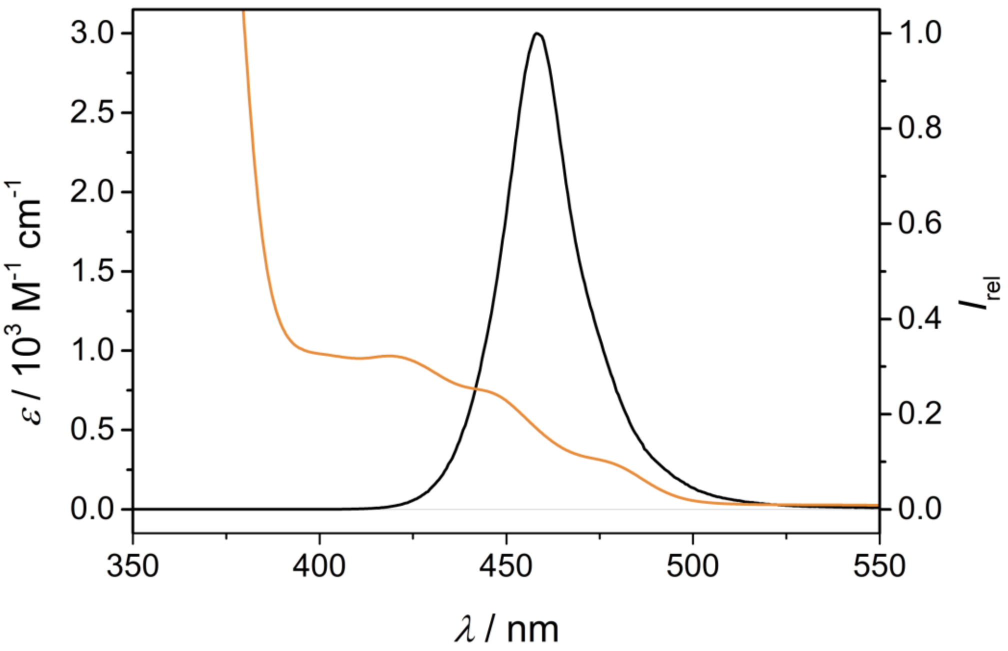

Supplementary Figure 2. UV/Vis spectrum of **[Cr(dmcbpy)$_3$][BF$_4$]$_3$** (1 mM) in acetonitrile (orange) and emission spectrum of the Aldrich® Micro Photochemical Reactor, blue LED lights (black).

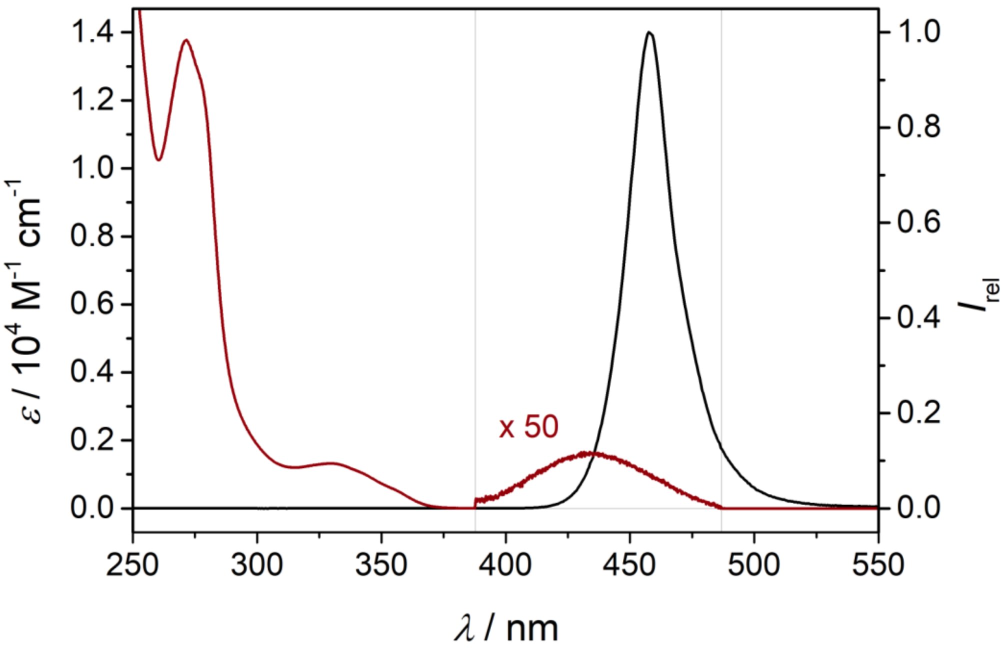

Supplementary Figure 3. UV/Vis spectrum of **$[Cr(tpe)_2][BF_4]_3$** (1 mM) in acetonitrile (red) and emission spectrum of the Aldrich® Micro Photochemical Reactor, blue LED lights (black).

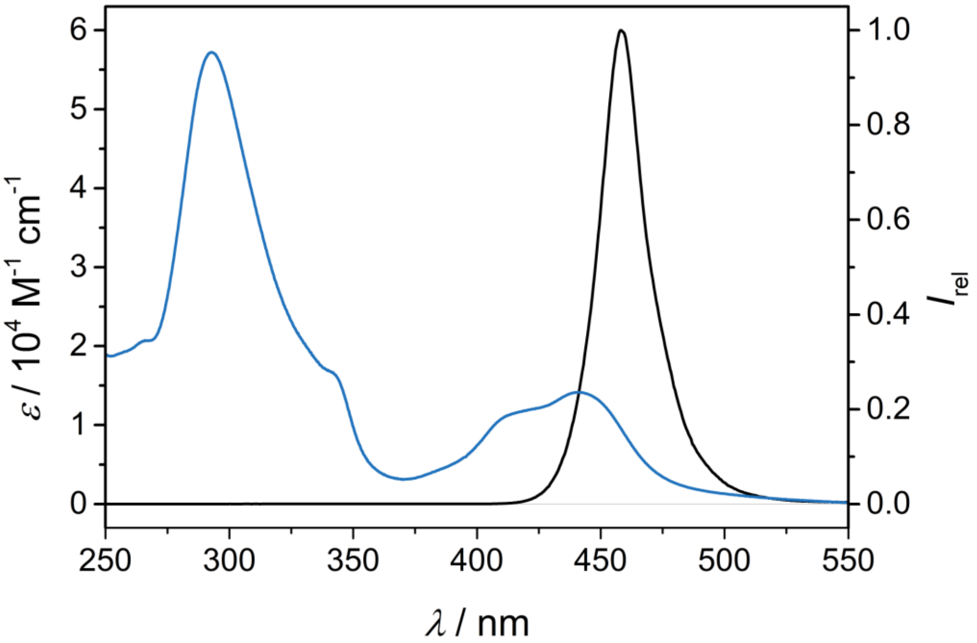

Supplementary Figure 4. UV/Vis spectrum of **$[Ru(bpz)_3][PF_6]_2$** (0.01 mM) in acetonitrile (blue) and emission spectrum of the Aldrich® Micro Photochemical Reactor, blue LED lights (black).

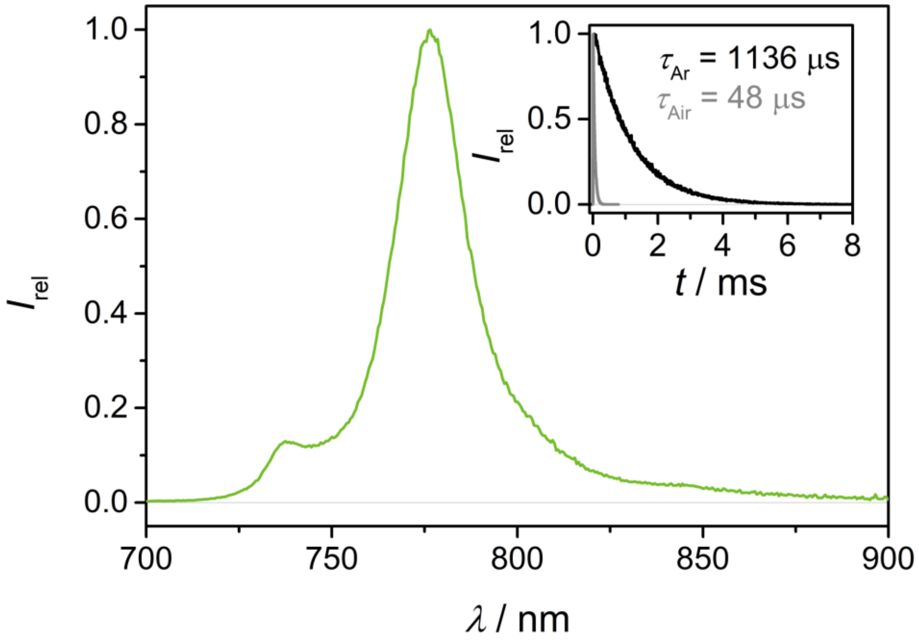

Supplementary Figure 5. Emission spectrum of **[Cr(ddpd)$_2$][BF$_4$]$_3$** (0.1 mM) in acetonitrile after excitation at 435 nm. Inset: emission decay curves under deaerated (black) and aerated (grey) conditions.

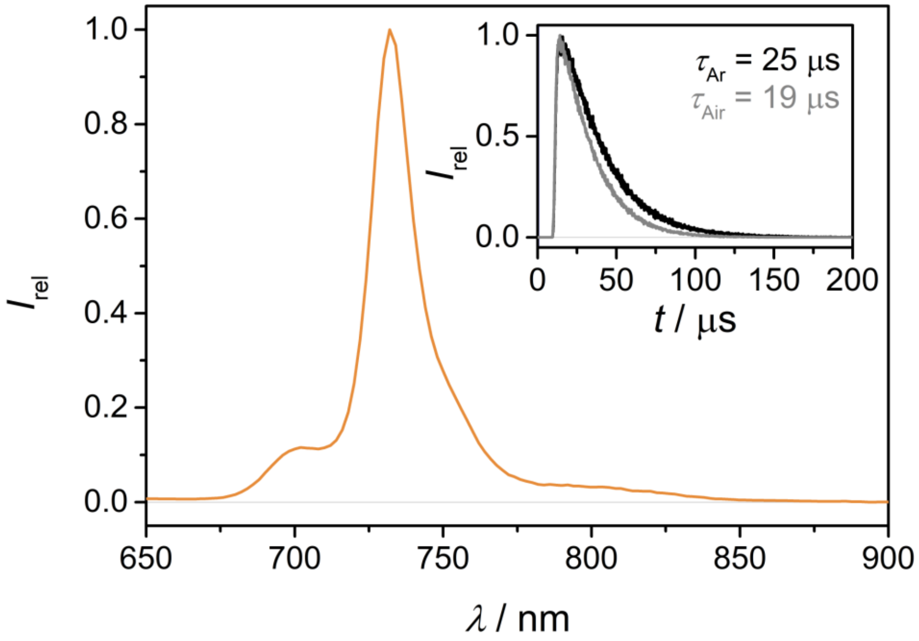

Supplementary Figure 6. Emission spectrum of **[Cr(dmcbpy)$_3$][BF$_4$]$_3$** (1 mM) in acetonitrile after excitation at 480 nm. Inset: emission decay curves under deaerated (black) and aerated (grey) conditions.

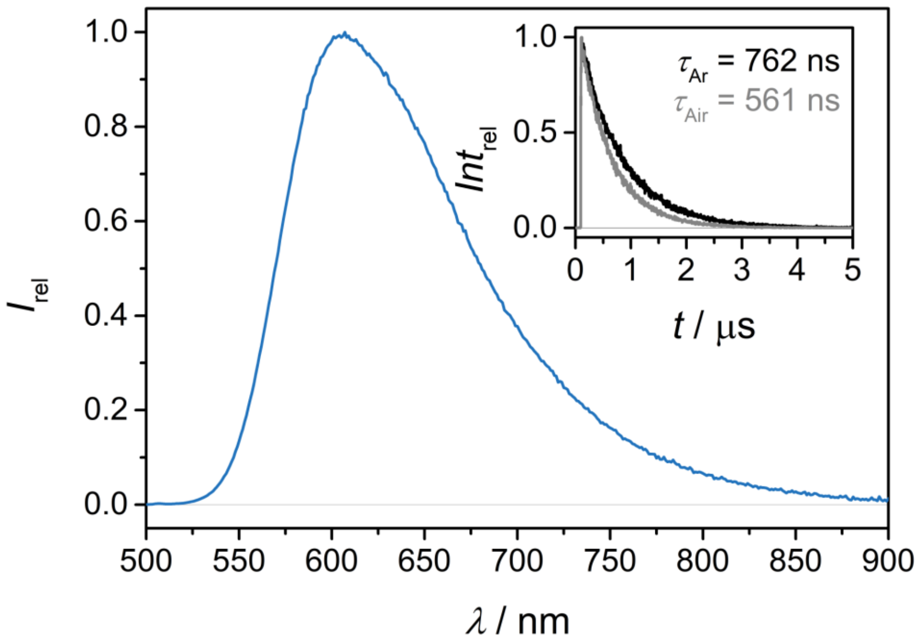

Supplementary Figure 7. Emission spectrum of **$[Ru(bpz)_3][PF_6]_2$** (0.01 mM) in acetonitrile after excitation at 440 nm. Inset: emission decay curves under deaerated (black) and aerated (grey) conditions.

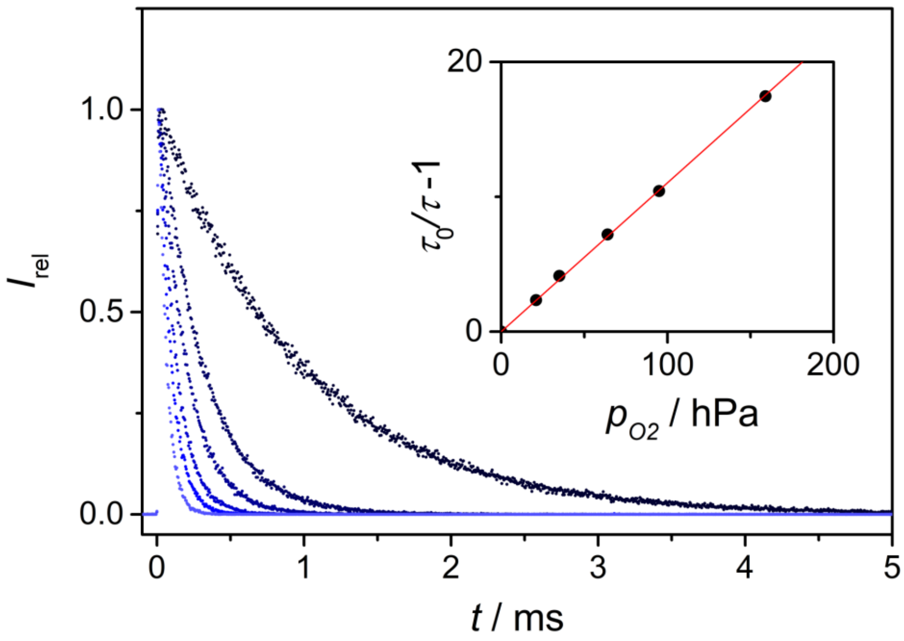

Supplementary Figure 8. Quenching of **$*[Cr(ddpd)_2]^{3+}$** by oxygen. Main plot, emission decay traces recorded at 776 nm following excitation (435 nm, 2 µs pulse duration) of a **$[Cr(ddpd)_2][BF_4]_3$** (0.1 mM) solution in deaerated acetonitrile with variable oxygen partial pressure p_{O2} (0 hPa, 21 hPa, 35 hPa, 64 hPa, 95 hPa, 159 hPa). Inset, corresponding Stern-Volmer plot with best-fit function overlaid on the data.

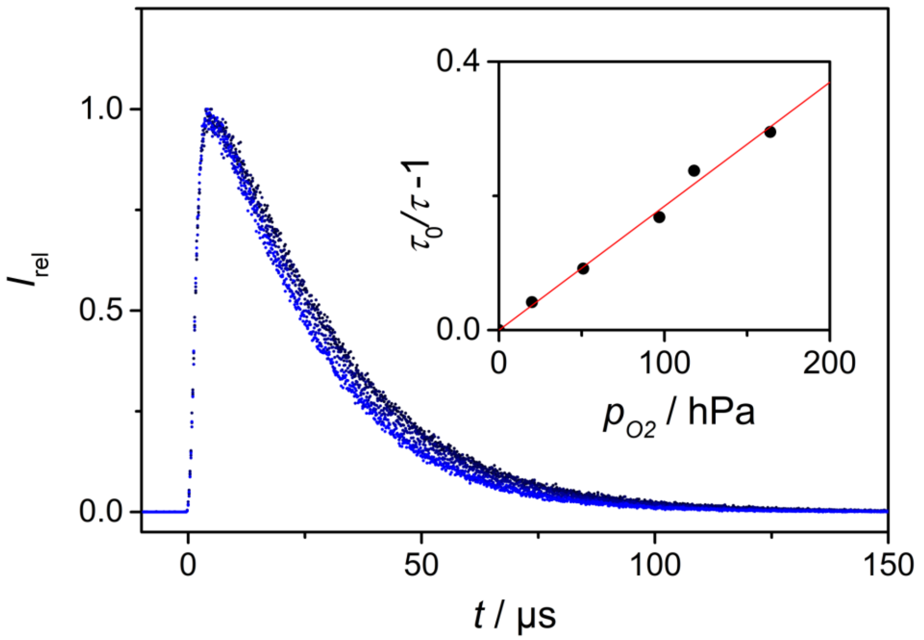

Supplementary Figure 9. Quenching of ***[Cr(dmcbpy)$_3$]$^{3+}$** by oxygen. Main plot, emission decay traces recorded at 733 nm following excitation (480 nm, 2 µs pulse duration) of a **[Cr(dmcbpy)$_3$][BF$_4$]$_3$** (0.5 mM) solution in deaerated acetonitrile with variable oxygen partial pressure p_{O2} (0 hPa, 20 hPa, 51 hPa, 97 hPa, 118 hPa, 164 hPa). Inset, corresponding Stern-Volmer plot with best-fit function overlaid on the data.

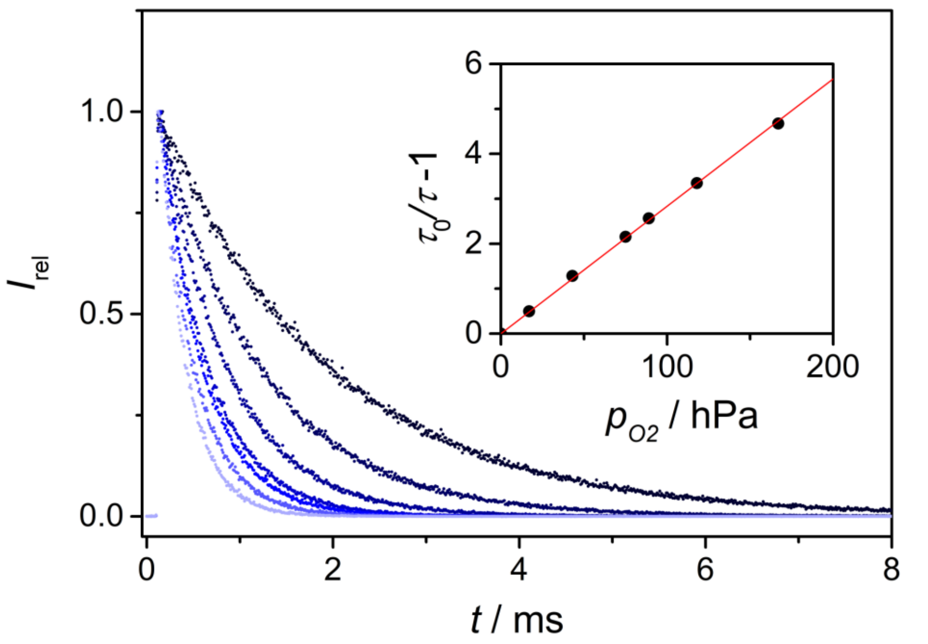

Supplementary Figure 10. Quenching of ***[Cr(tpe)$_2$]$^{3+}$** by oxygen. Main plot, emission decay traces recorded at 742 nm following excitation (435 nm, 2 µs pulse duration) of a **[Cr(tpe)$_2$][BF$_4$]$_3$** (1 mM) solution in deaerated acetonitrile with variable oxygen partial pressure p_{O2} (0 hPa, 17 hPa, 43 hPa, 75 hPa, 89 hPa, 118 hPa, 167 hPa). Inset, corresponding Stern-Volmer plot with best-fit function overlaid on the data.

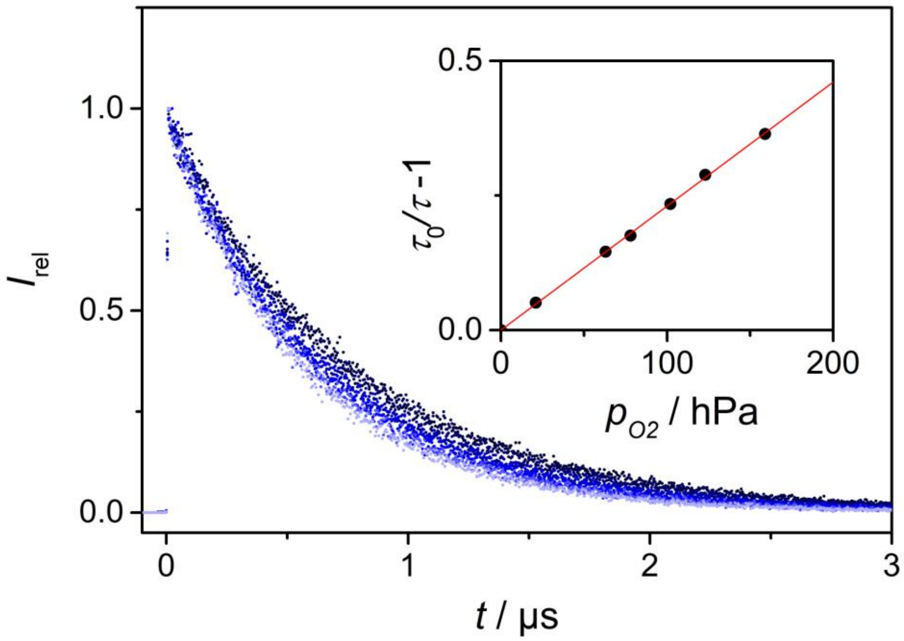

Supplementary Figure 11. Quenching of **$*[Ru(bpz)_3]^{2+}$** by oxygen. Main plot, emission decay traces recorded at 606 nm following laser excitation (440 nm) of a **$[Ru(bpz)_3][PF_6]_2$** (0.01 mM) solution in deaerated acetonitrile with variable oxygen partial pressure p_{O2} (0 hPa, 21 hPa, 63 hPa, 78 hPa, 102 hPa, 123 hPa, 159 hPa). Inset, corresponding Stern-Volmer plot with best-fit function overlaid on the data.

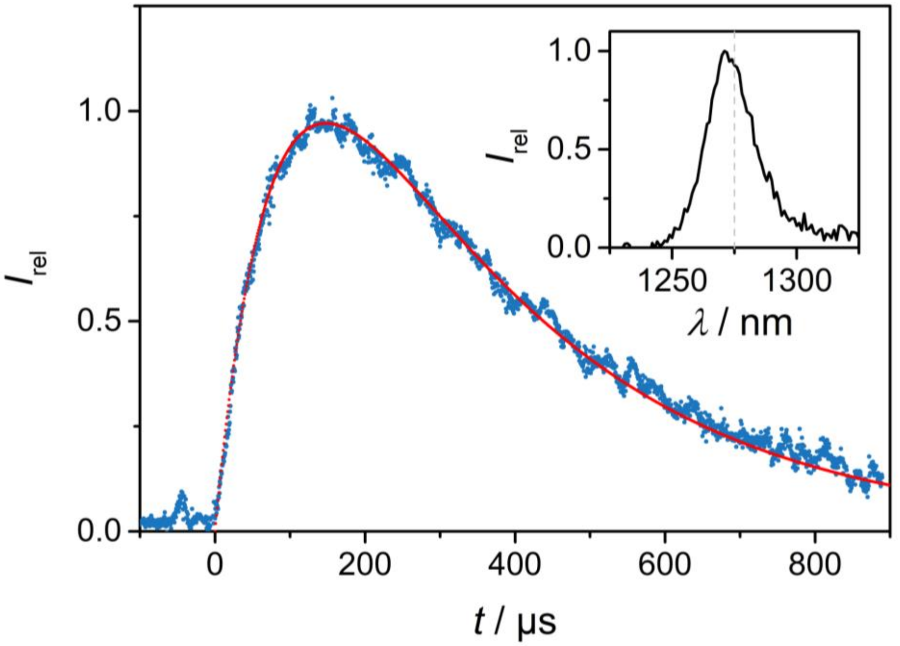

Supplementary Figure 12. $^1O_2$ emission upon excitation of 1 mM **$[Cr(tpe)_2][BF_4]_3$** in air-saturated acetonitrile with a xenon-lamp at 355 nm. Main plot, decay trace of the emission at 1275 nm (blue) with the best-fit function (red dots) overlaid on the data. Inset, corresponding emission spectrum. For further explanations, see text.

Kinetic modeling of the $^1O_2$ decay trace (Supplementary Figure 12)

$^1O_2$ forms via Dexter energy transfer from ***PC**$^{3+}$ to $^3O_2$ and decays mainly via non-radiative relaxation with the radiative relaxation neglected:

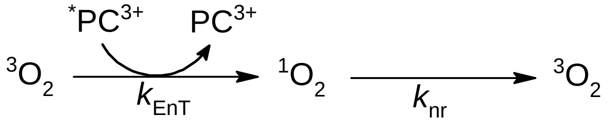

(S-1)

The processes follow second- (pseudo-first-order with [$^3O_2$] constant) and first-order kinetics, respectively, so that the rate law for the $^1O_2$ concentration [$^1O_2$] can be written as:

$$\frac{d[\,^1O_2]}{dt} = k_{EnT}[\,^3O_2][\,^*PC^{3+}] - k_{nr}[\,^1O_2] \qquad \text{(S-2)}$$

For the first-order decay of the emissive ***PC**$^{3+}$ with the rate constant k_{lum} and the initial concentration [***PC**$^{3+}$]$_0$ the integrated rate law equals:

$$[\,^*\boldsymbol{PC}^{\boldsymbol{3+}}] = [\,^*\boldsymbol{PC}^{\boldsymbol{3+}}]_0\, e^{-k_{lum}t} \qquad \text{(S-3)}$$

Insertion of S-3 into S-2 and subsequent integration gives the integrated rate law of [$^1O_2$]:

$$[\,^1O_2] = \left(\frac{[\,^*\boldsymbol{PC}^{\boldsymbol{3+}}]_0 \cdot k_{EnT} \cdot [\,^3O_2]}{k_{lum} + k_{EnT} \cdot [\,^3O_2] - k_{nr}}\right) \cdot \left(e^{-k_{nr}t} - e^{-(k_{lum} + k_{EnT} \cdot [\,^3O_2])t}\right) \qquad \text{(S-4)}$$

The constants k_{EnT} [$^3O_2$] and (k_{lum} + k_{EnT} [$^3O_2$]) can be calculated from the independently determined lifetimes of ***[Cr(tpe)$_2$]$^{3+}$** in deaerated (1965 μs) and air-saturated acetonitrile (304 μs). As k_{nr} is known from the literature[12] (k_{nr} = 1.2×10^4 s^{-1}) the scaling factor [***PC**$^{3+}$]$_0$ remains as the only degree of freedom in the fit overlapped on the data in Supplementary Figure 12.

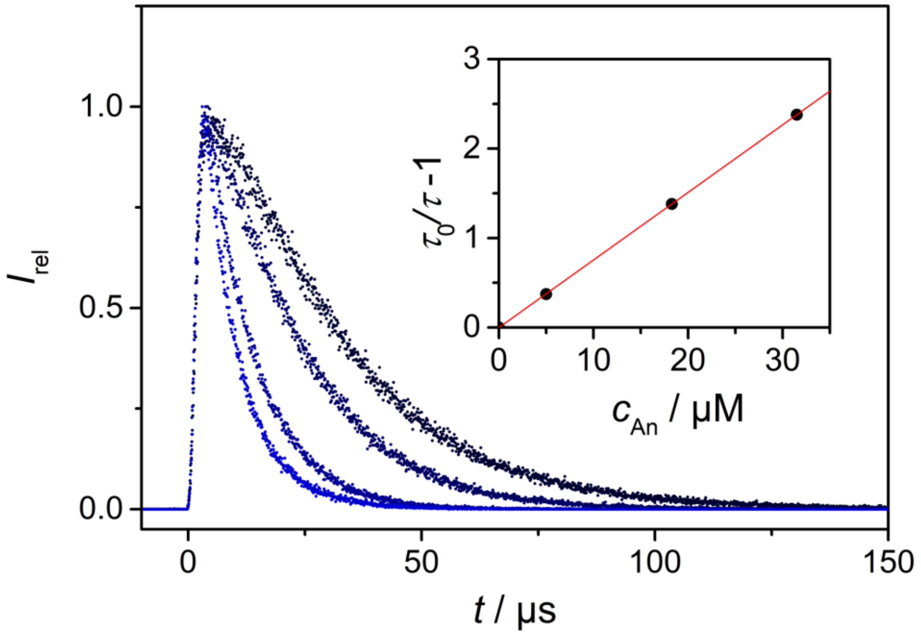

Supplementary Figure 13. Reductive quenching of ***[Cr(dmcbpy)$_3$]$^{3+}$** by ***tAn***. Main plot, emission decay traces recorded at 733 nm following excitation (480 nm, 2 μs pulse duration) of a **[Cr(dmcbpy)$_3$][BF$_4$]$_3$** (0.5 mM) solution in deaerated acetonitrile with variable amounts of ***tAn*** (0 mM, 5.0 mM, 18.3 mM, 31.5 mM). Inset, corresponding Stern-Volmer plot with best-fit function overlaid on the data.

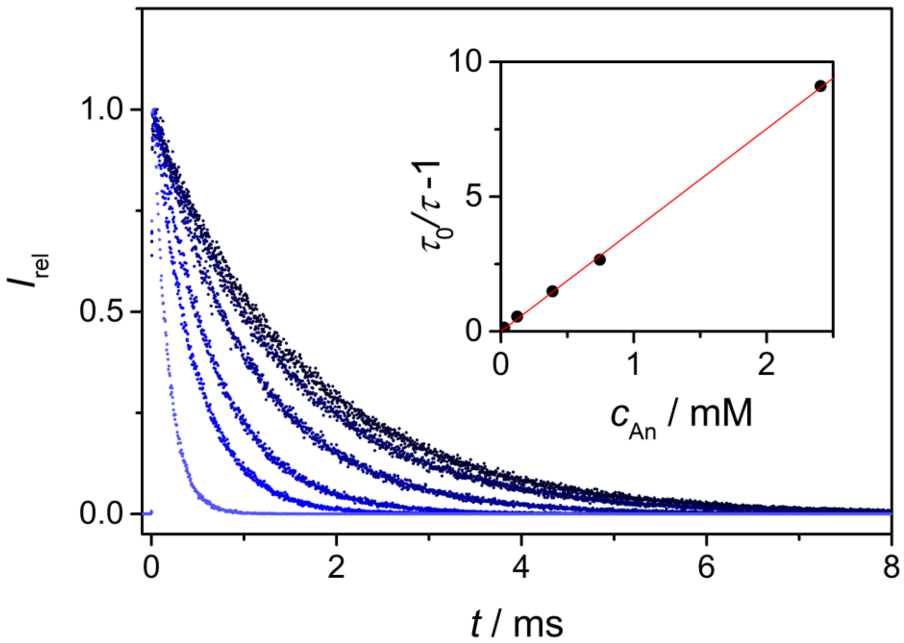

Supplementary Figure 14. Reductive quenching of **$*[Cr(tpe)_2]^{3+}$** by ***t*An**. Main plot, emission decay traces recorded at 742 nm following excitation (435 nm, 2 μs pulse duration) of a **$[Cr(tpe)_2][BF_4]_3$** (1 mM) solution in deaerated acetonitrile with variable amounts of *t*An (0 mM, 0.02 mM, 0.12 mM, 0.39 mM, 0.74 mM, 2.41 mM). Inset, corresponding Stern-Volmer plot with best-fit function overlaid on the data.

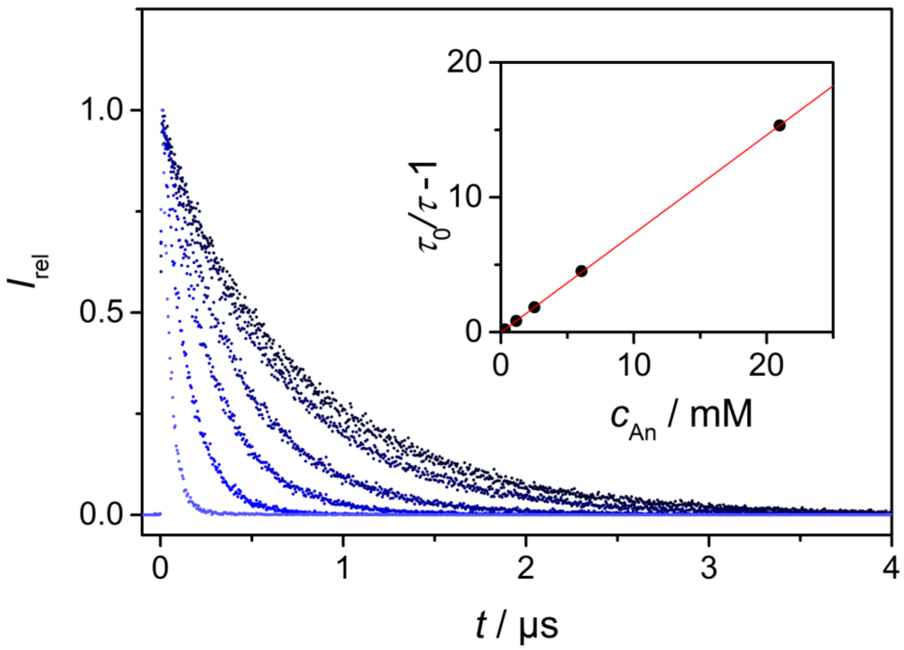

Supplementary Figure 15. Reductive quenching of **$*[Ru(bpz)_3]^{2+}$** by ***t*An**. Main plot, emission decay traces recorded at 606 nm following laser excitation (440 nm) of a **$[Ru(bpz)_3][PF_6]_2$** (0.01 mM) solution in deaerated acetonitrile with variable amounts of *t*An (0 mM, 0.3 mM, 1.2 mM, 2.5 mM, 6.1 mM, 21 mM). Inset, corresponding Stern-Volmer plot with best-fit function overlaid on the data.

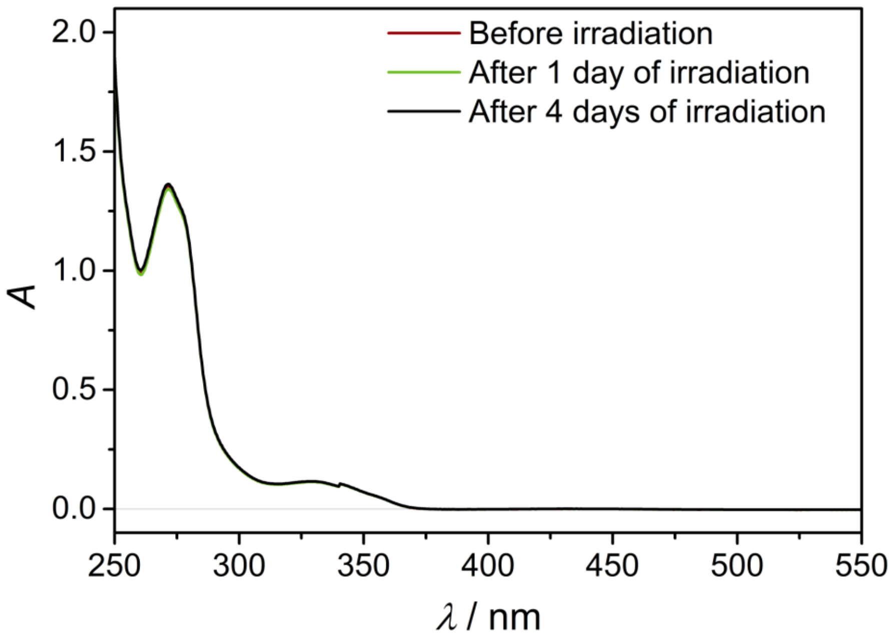

Supplementary Figure 16. UV/Vis spectra of **[Cr(tpe)$_2$][BF$_4$]$_3$** in deaerated acetonitrile before (red), after 1 day (green) and after 4 days (black) of irradiation (460 nm).

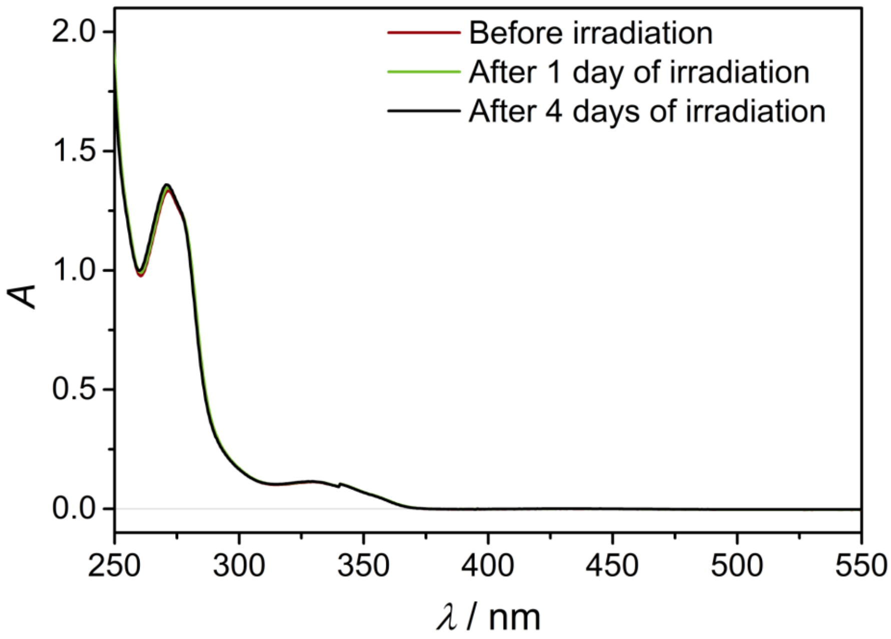

Supplementary Figure 17. UV/Vis spectra of **[Cr(tpe)$_2$][BF$_4$]$_3$** in aerated acetonitrile before (red), after 1 day (green) and after 4 days (black) of irradiation (460 nm).

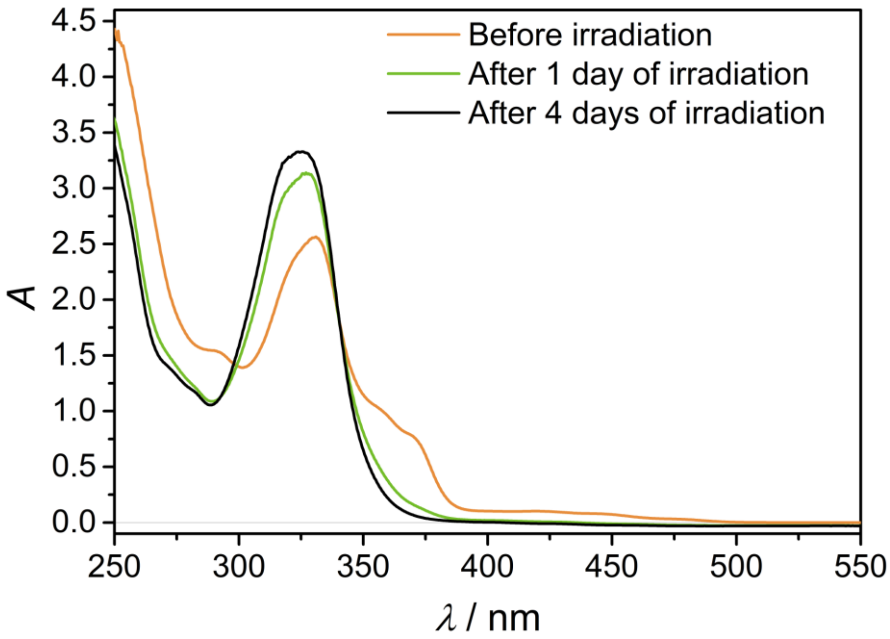

Supplementary Figure 18. UV/Vis spectra of **[Cr(dmcbpy)$_3$][BF$_4$]$_3$** in deaerated acetonitrile before (orange), after 1 day (green) and after 4 days (black) of irradiation (460 nm).

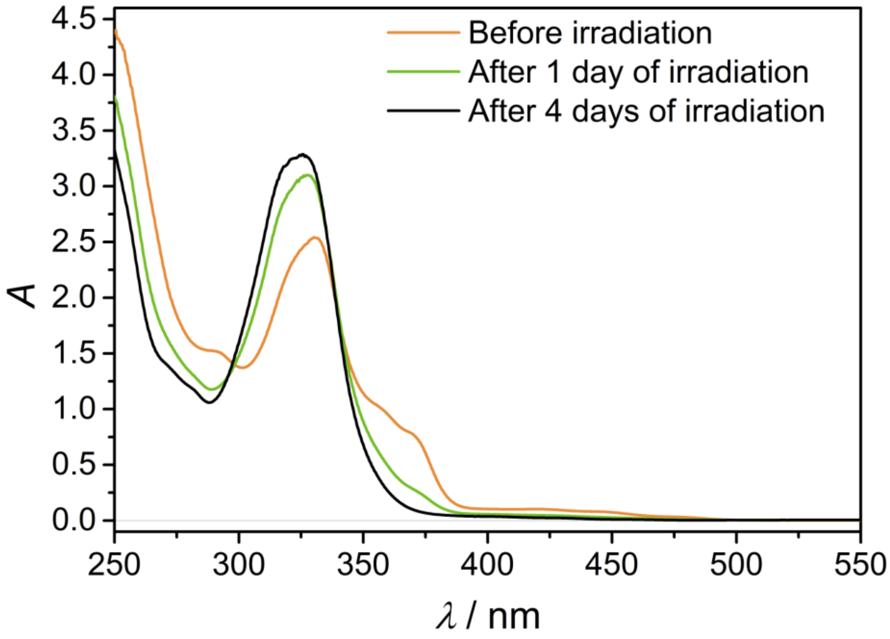

Supplementary Figure 19. UV/Vis spectra of **[Cr(dmcbpy)$_3$][BF$_4$]$_3$** in aerated acetonitrile before (orange), after 1 day (green) and after 4 days (black) of irradiation (460 nm).

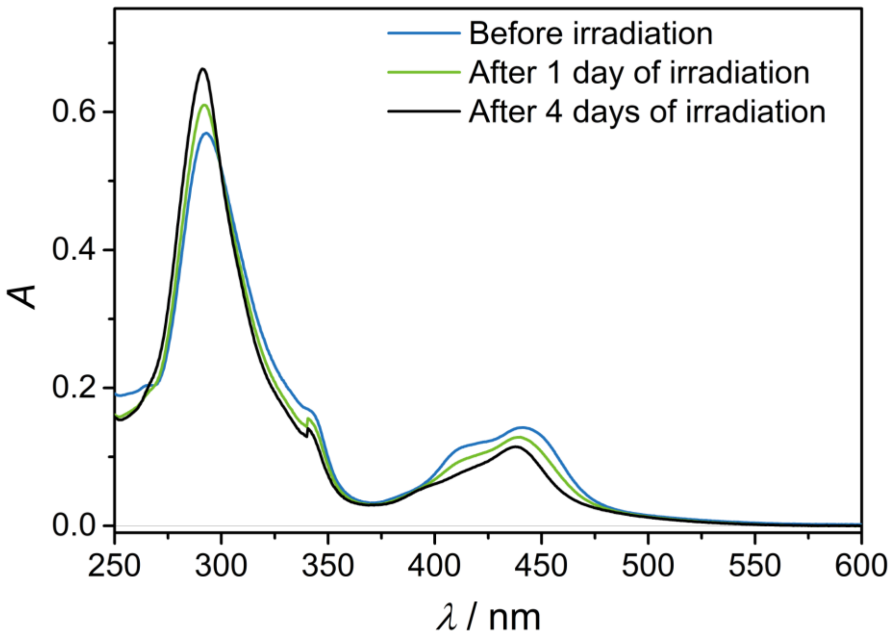

Supplementary Figure 20. UV/Vis spectra of **$[Ru(bpz)_3][PF_6]_2$** in deaerated acetonitrile before (blue), after 1 day (green) and after 4 days (black) of irradiation (460 nm).

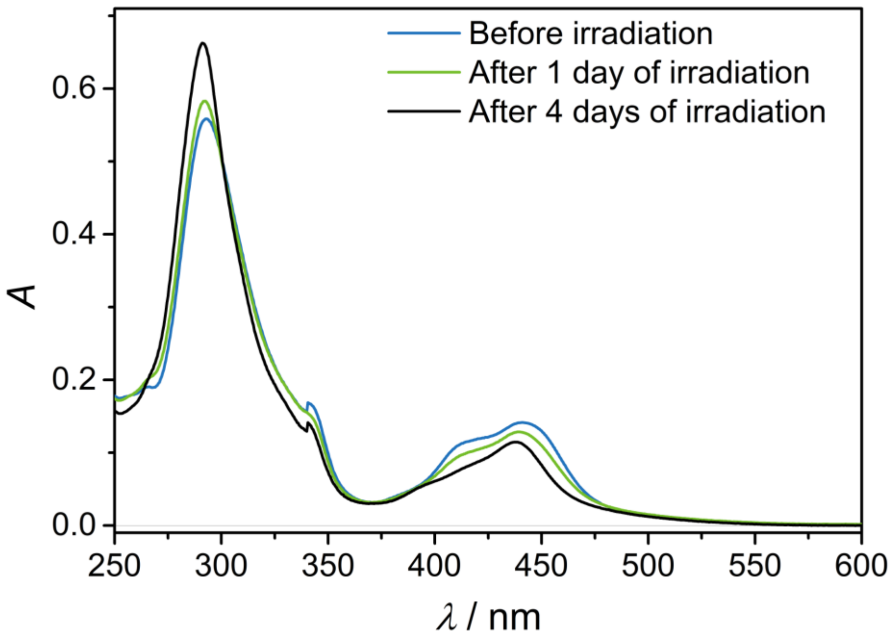

Supplementary Figure 21. UV/Vis spectra of **$[Ru(bpz)_3][PF_6]_2$** in aerated acetonitrile before (blue), after 1 day (green) and after 4 days (black) of irradiation (460 nm).

Supplementary Table 2. TD-DFT (CPCM(acetonitrile)-UB3LYP-D3BJ-ZORA/def2-TZVPP) calculated lowest-energy spin-allowed transitions for the quartet ground state[13] and the lowest doublet state. Hydrogen atoms omitted. Difference electron densities plotted with an isosurface value of 0.004 a.u.; purple = electron depletion; orange = electron gain. Transition dipole moments given in parentheses. Other low-energy transitions possess oscillator strengths below 10^{-8}, and hence these are not given.

quartet ground state:<br>transitions with LMCT/ILCT character			lowest doublet state:<br>transitions with LMCT/ILCT character		
#7	326 nm<br>(0.0044)	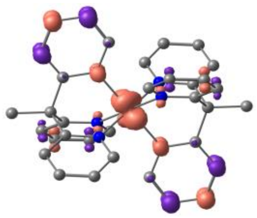	#9	375 nm<br>(0.0016)	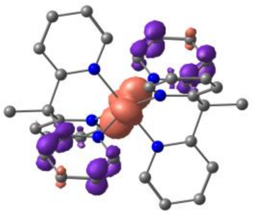
#8	326 nm<br>(0.0043)	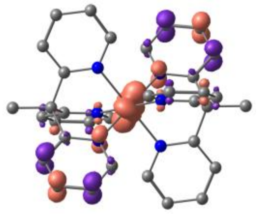	#10	373 nm<br>(0.0034)	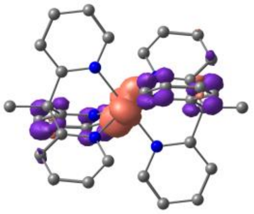
#9	322 nm<br>(0.0027)	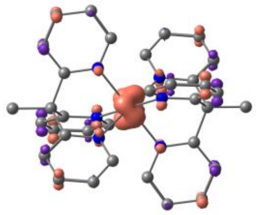	#13	372 nm<br>(0.0021)	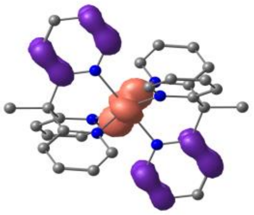
			#14	345 nm<br>(<<0.001)	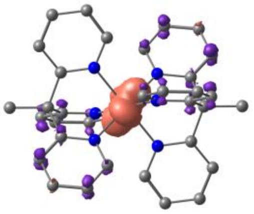
			#15	345 nm<br>(0.0045)	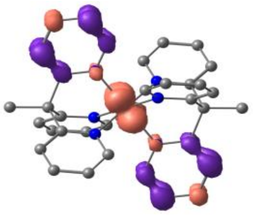
			#16	345 nm<br>(<<0.001)	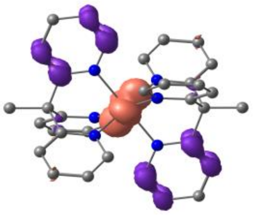

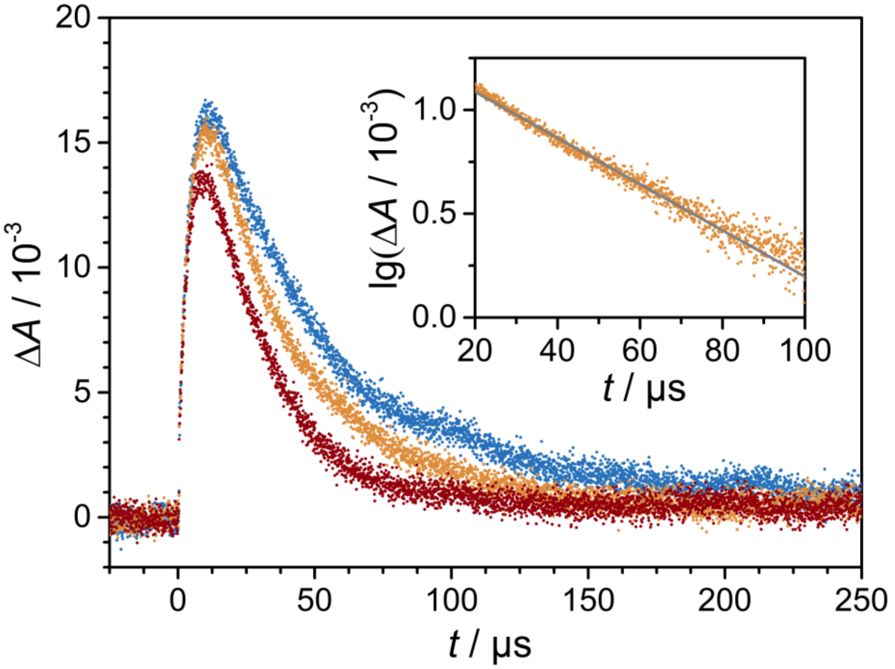

Supplementary Figure 22. Repeated (eight times) measurement of the decay of ***t*An**$^{\bullet+}$ (detection at 606 nm) following laser excitation at 355 nm of a **[Cr(tpe)$_2$][BF$_4$]$_3$** (0.35 mM) solution in *deaerated* acetonitrile quenched by 0.1 M ***t*An**. Main plot, experimental decay traces for the first (blue), the third (orange) and the eighth (red) iteration. Inset: corresponding linearization of a first-order process for the third iteration (orange) with best-fit function (gray line) overlaid on the data.

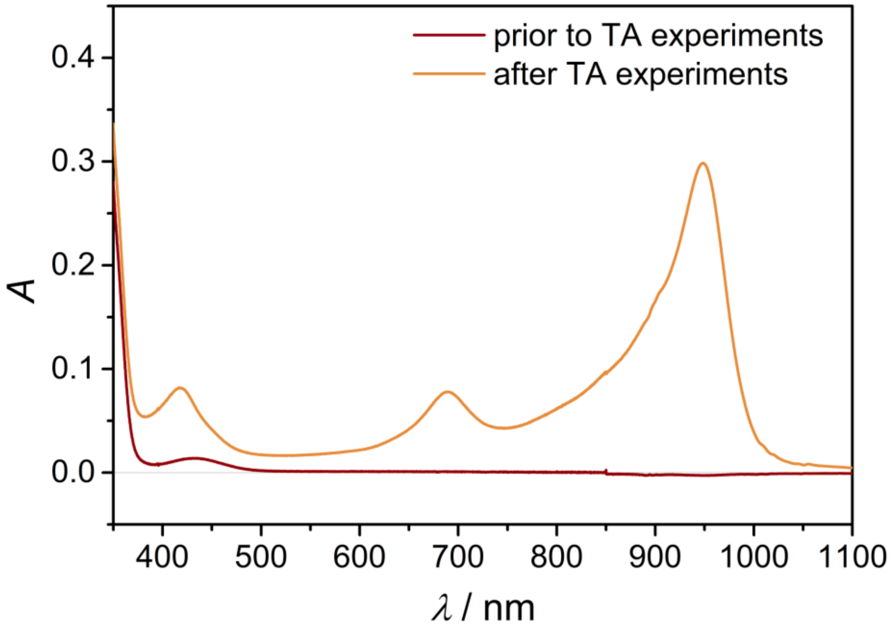

Supplementary Figure 23. UV/Vis/NIR spectrum of the solution (Figure 22) prior to (red) and after (orange) TA experiments.

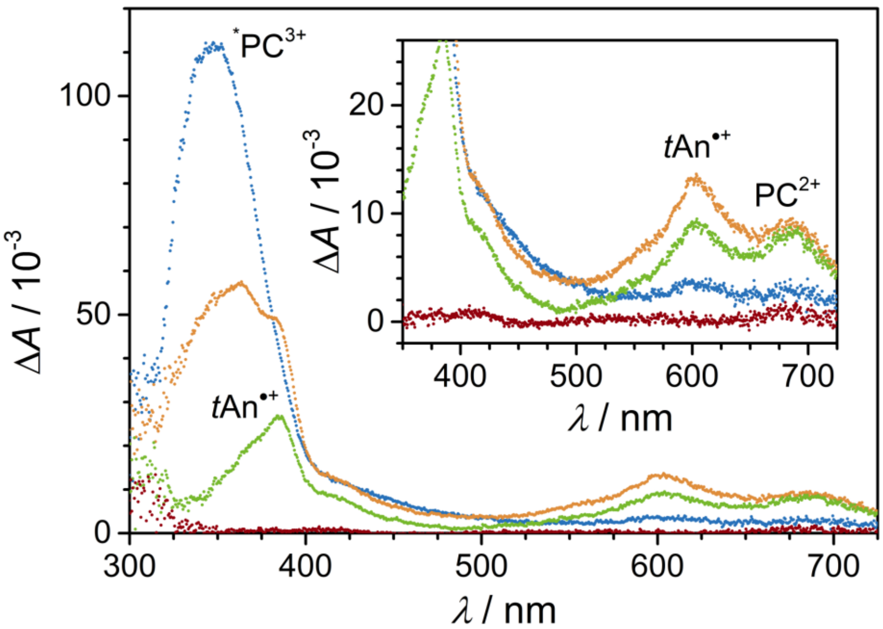

Supplementary Figure 24. ns-TA spectra illustrating the evolution and the decay of ***PC^{3+}*** (345 nm), ***t*An$^{•+}$** (385 nm, 606 nm) and **PC^{2+}** (690 nm) following laser excitation at 355 nm of a **[Cr(tpe)$_2$][BF$_4$]$_3$** (0.35 mM) solution in *deaerated* acetonitrile with 0.1 M ***t*An** and 0.5 M **DMB**. The transient absorption spectra are time-integrated over 100 ns and taken at a time delay of 50 ns (blue), 5 µs (orange), 50 µs (green) and 500 µs (red) relative to the excitation pulse.

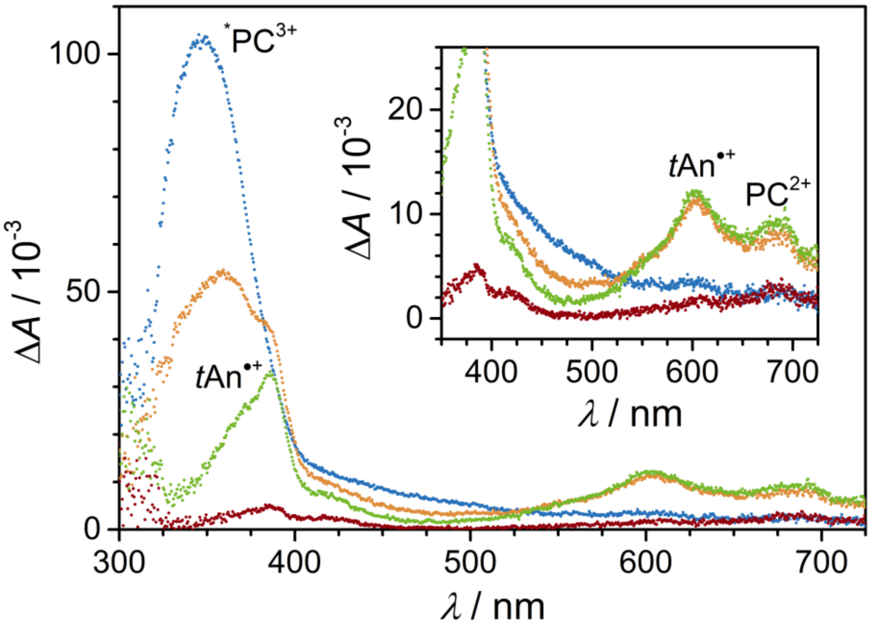

Supplementary Figure 25. ns-TA spectra illustrating the evolution and the decay of ***PC^{3+}*** (345 nm), ***t*An$^{•+}$** (385 nm, 606 nm) and **PC^{2+}** (690 nm) following laser excitation at 355 nm of a **[Cr(tpe)$_2$][BF$_4$]$_3$** (0.35 mM) solution in *air-saturated* acetonitrile with 0.1 M ***t*An** and 0.5 M **DMB**. The transient absorption spectra are time-integrated over 100 ns and taken at a time delay of 50 ns (blue), 5 µs (orange), 50 µs (green) and 500 µs (red) relative to the excitation pulse.

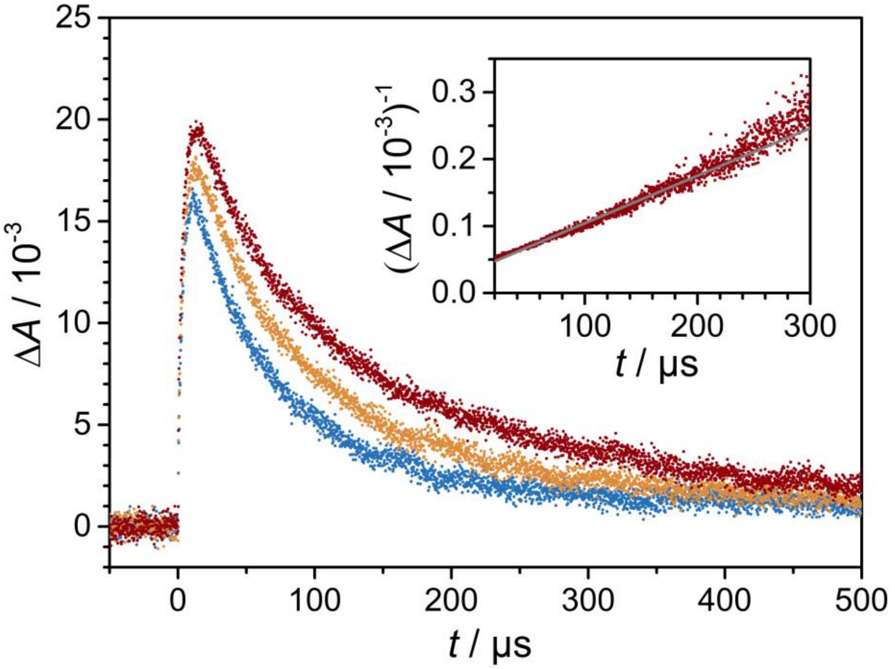

Supplementary Figure 26. Repeated (eight times) measurement of the decay of ***t*An**$^{•+}$ (detection at 606 nm) following laser excitation at 355 nm of a **[Cr(tpe)$_2$][BF$_4$]$_3$** (0.35 mM) solution in *air-saturated* acetonitrile quenched by 0.1 M ***t*An**. Main plot, experimental decay traces for the first (blue), the third (orange) and the eighth (red) iteration. Inset: corresponding linearization of a second-order process for the eighth iteration (red) with best-fit function (gray line) overlaid on the data.

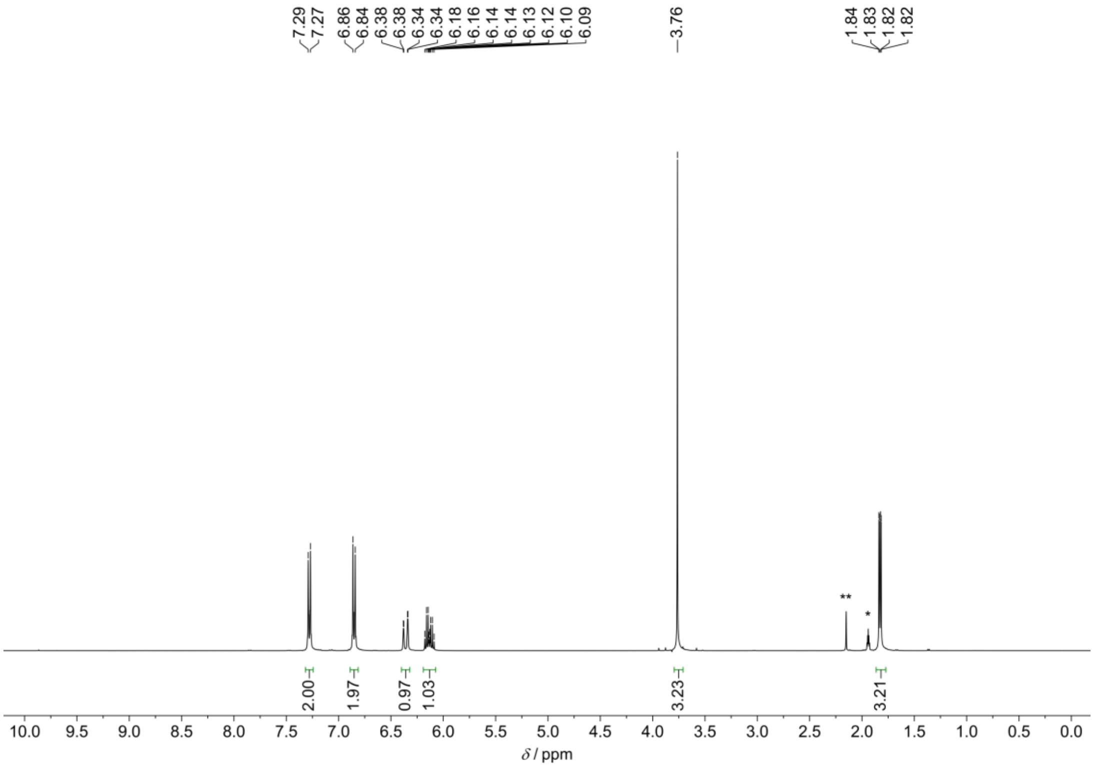

Supplementary Figure 27. ^{1}H NMR spectrum of ***t*An** in CD_3CN. * denotes acetonitrile solvent resonance, ** denotes water resonance.

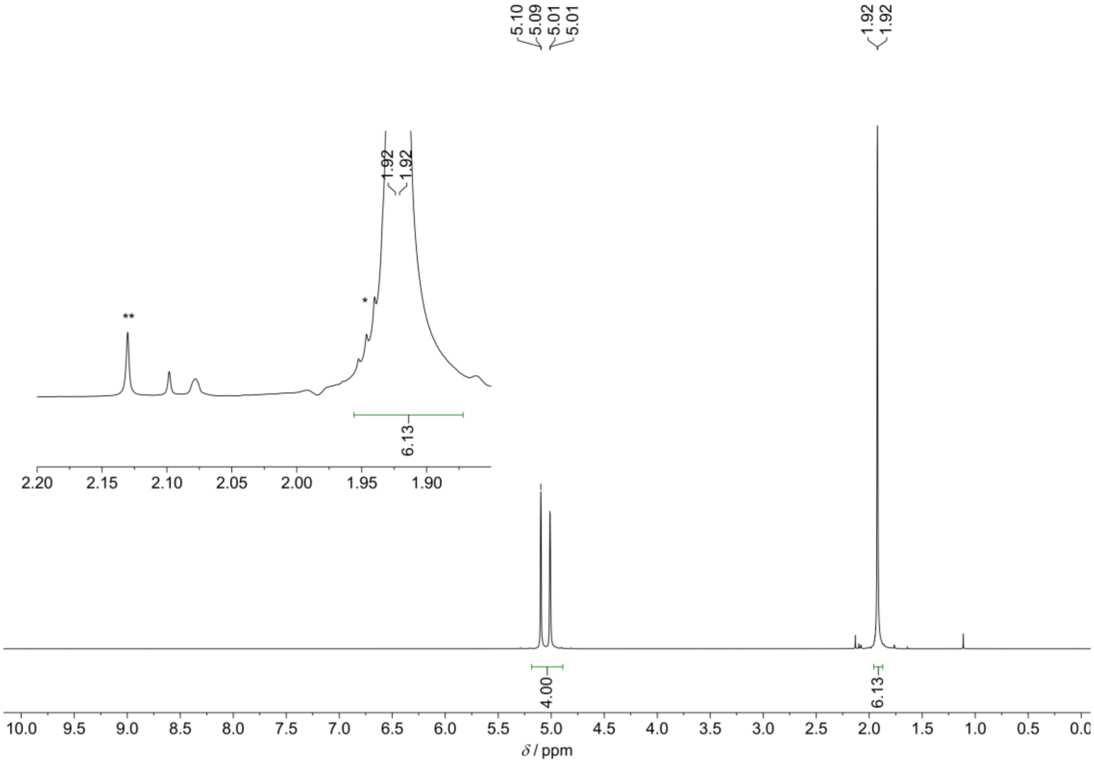

Supplementary Figure 28. ^{1}H NMR spectrum of **DMB** in CD_3CN. * denotes acetonitrile solvent resonance, ** denotes water resonance.

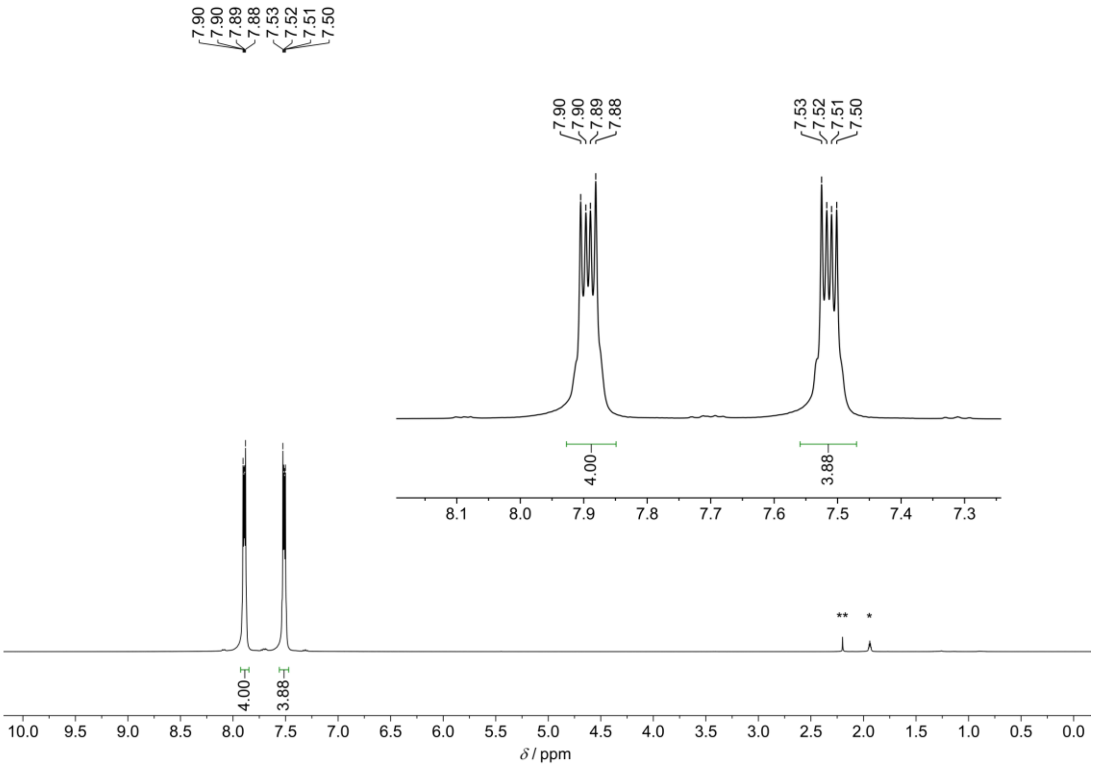

Supplementary Figure 29. ^{1}H NMR spectrum of **naphthalene** in CD_3CN. * denotes acetonitrile solvent resonance, ** denotes water resonance.

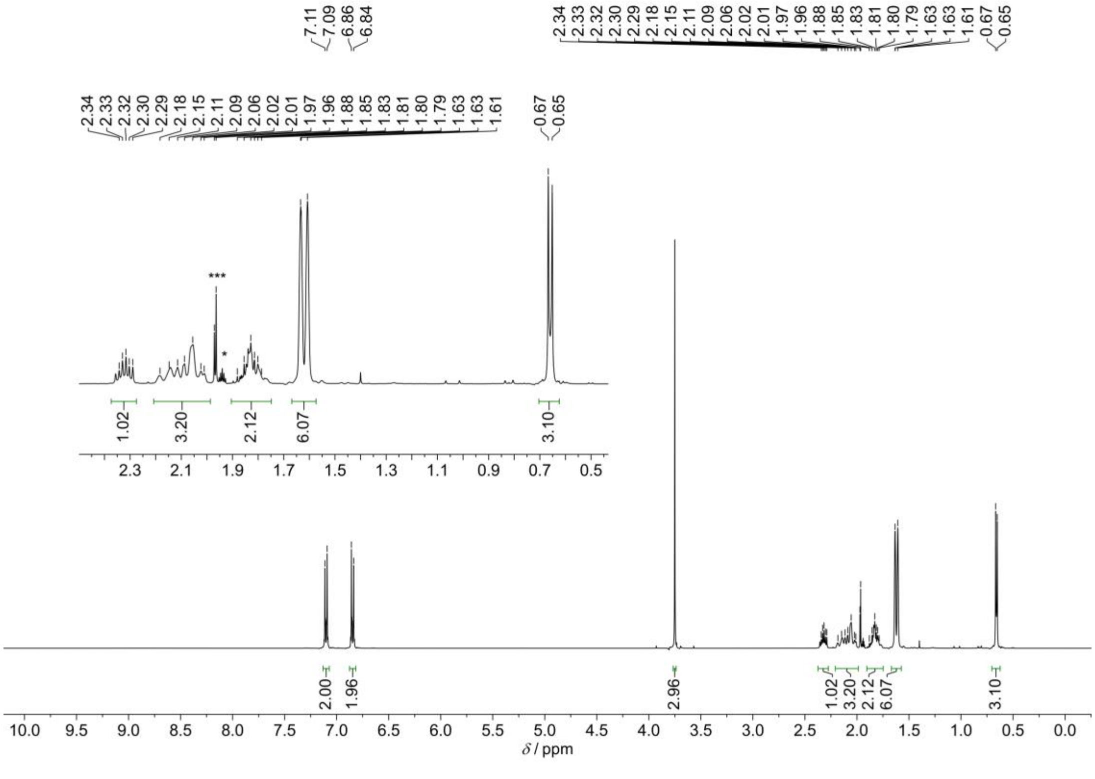

Supplementary Figure 30. ^{1}H NMR spectrum of the isolated product **DAP** in CD_3CN. * denotes acetonitrile solvent resonance, *** denotes a resonance of an unknown impurity.

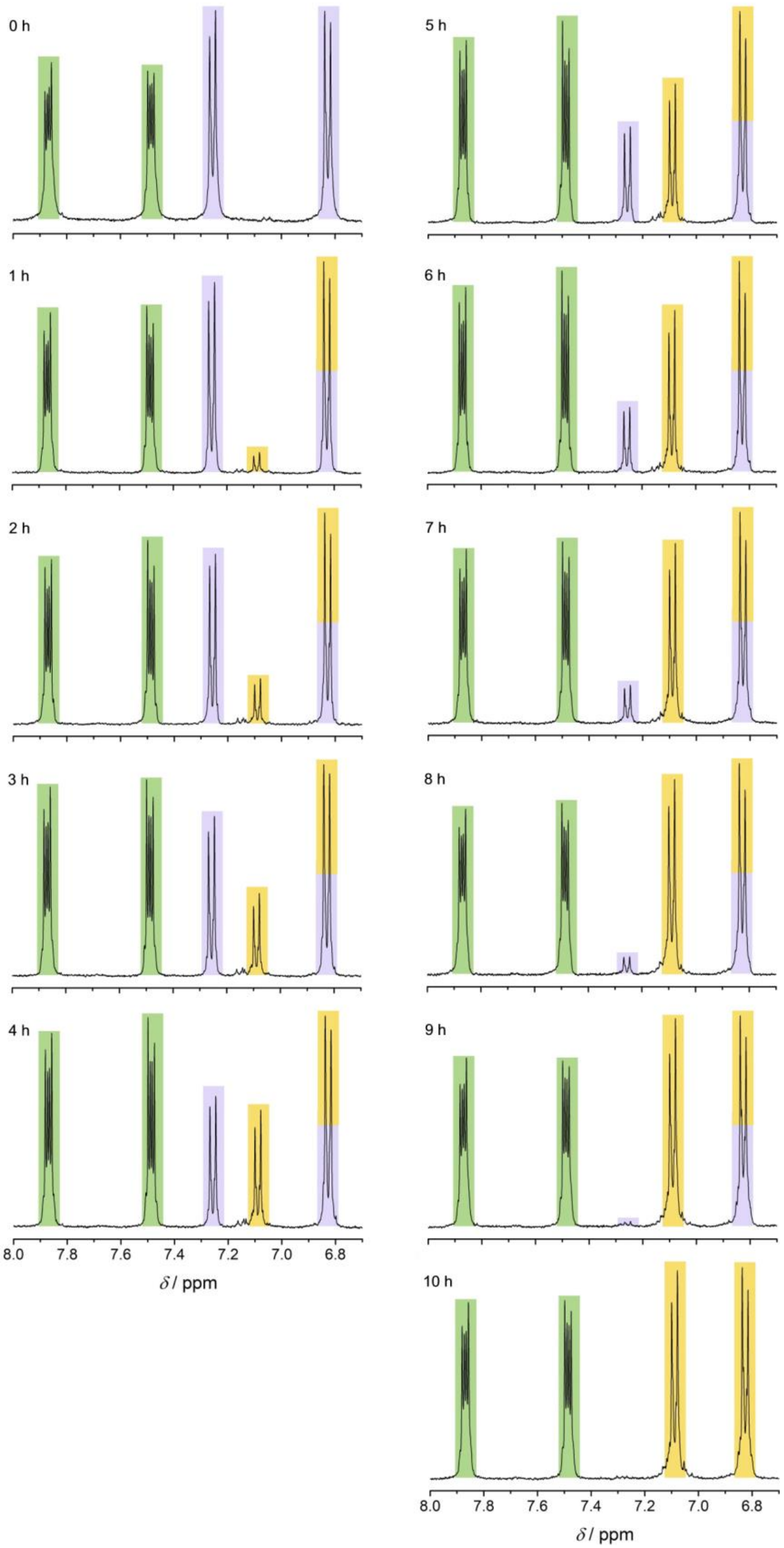

Supplementary Figure 31. Exemplary partial ^{1}H NMR spectra of the reaction using 100 mM ***t*An** (purple), 500 mM **DMB** and 0.2 mM **[Cr(tpe)$_2$][BF$_4$]$_3$** in acetonitrile with naphthalene (green) as internal standard with 460 nm light excitation. Product resonances of DAP highlighted in yellow.

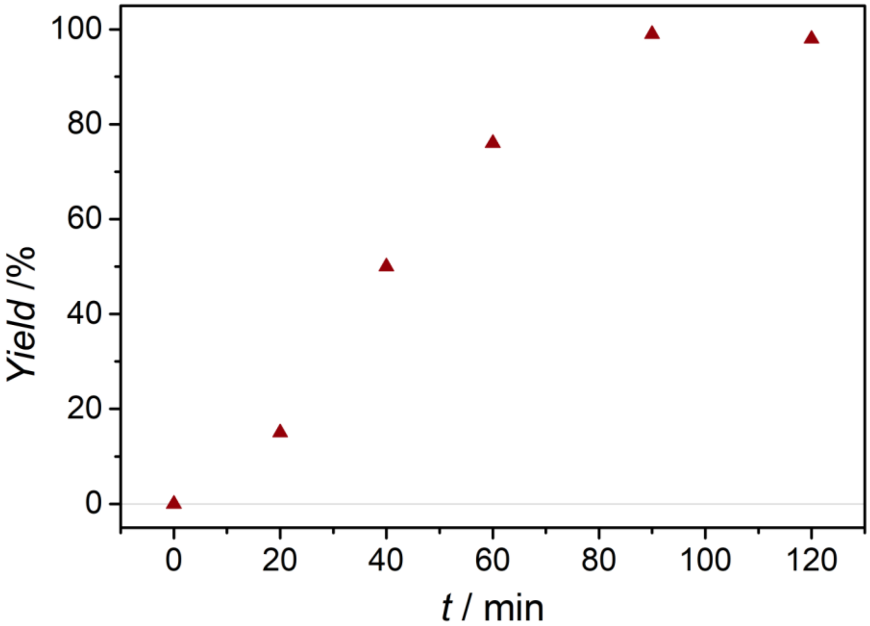

Supplementary Figure 32. Conversion vs. times plot of the photoredox catalyzed radical cation Diels−Alder cycloaddition of 100 mM ***t*An** and 500 mM **DMB** using **[Cr(tpe)$_2$]**$^{3+}$ (5 mM) in CH_3CN with 460 nm light excitation.

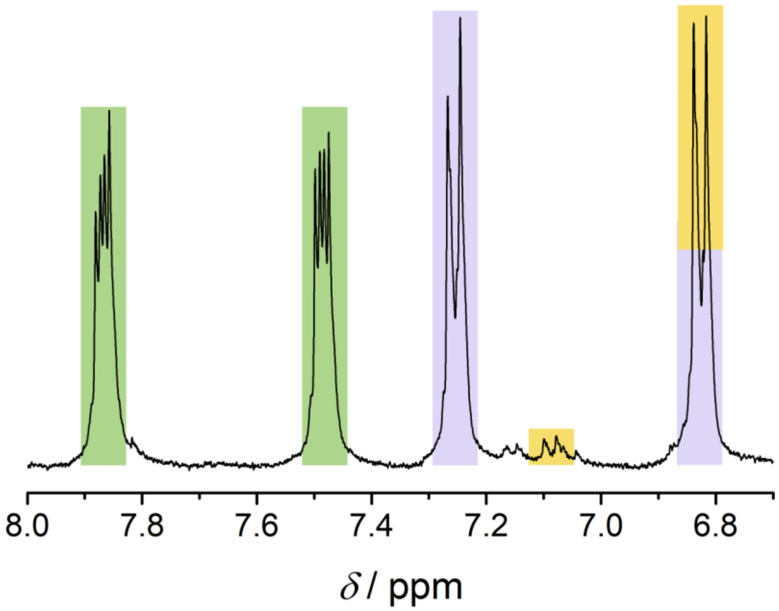

Supplementary Figure 33. Partial ^{1}H NMR spectrum of the reaction mixture 100 mM ***t*An** (purple), 500 mM **DMB** and 1 mM **[Cr(tpe)$_2$][BF$_4$]$_3$** in acetonitrile in the absence of air with 460 nm light excitation after 4 h using naphthalene (green) as internal standard. Product resonances of DAP highlighted in yellow.

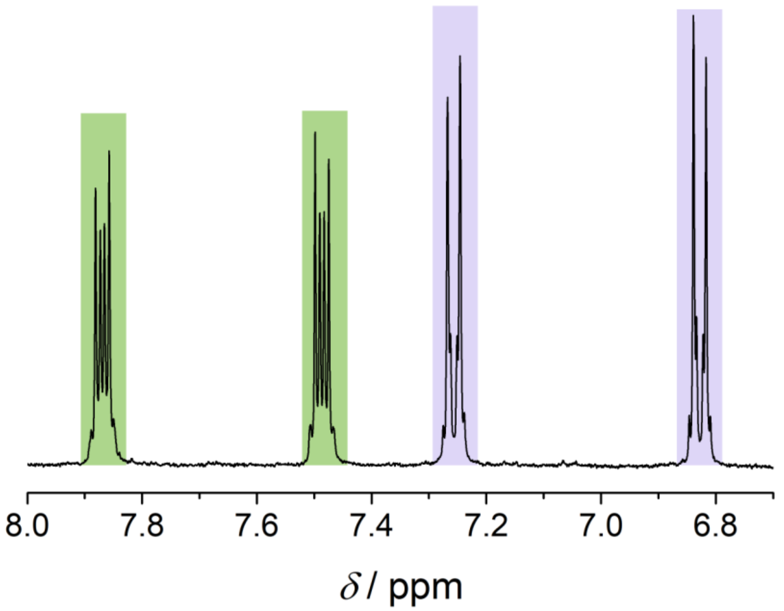

Supplementary Figure 34. Partial ^{1}H NMR spectrum of the reaction mixture 100 mM ***t*An** (purple), 500 mM **DMB** and 1 mM **[Cr(tpe)$_2$][BF$_4$]$_3$** in acetonitrile in the presence of air in the dark after 24 h using naphthalene (green) as internal standard.

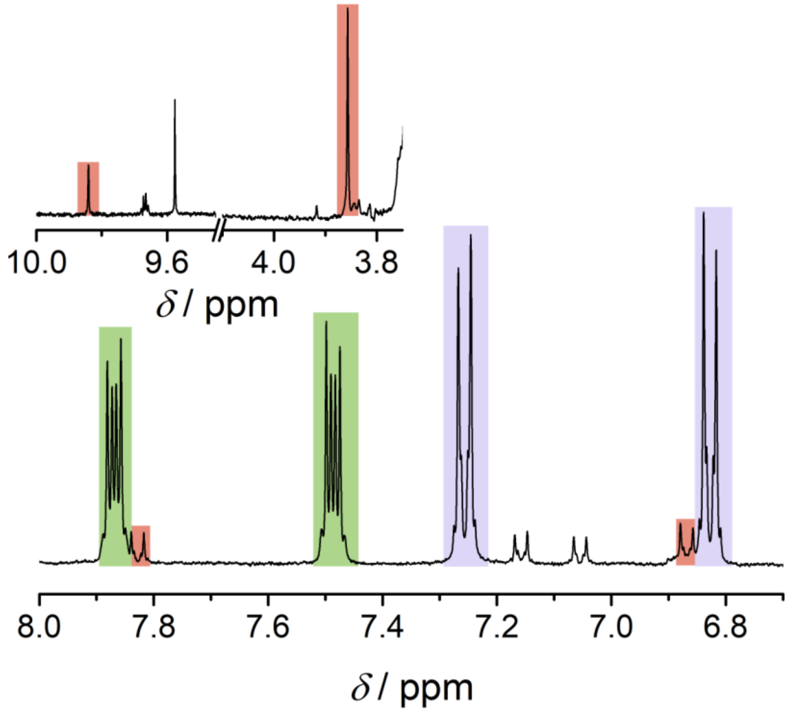

Supplementary Figure 35. Partial ^{1}H NMR spectrum of the reaction mixture 100 mM ***t*An** (purple) and 500 mM **DMB** without a photocatalyst in acetonitrile in the presence of air with 460 nm light excitation after 24 h using naphthalene (green) as internal standard. The weak resonances highlighted in red are assigned to 4-anisaldehyde.

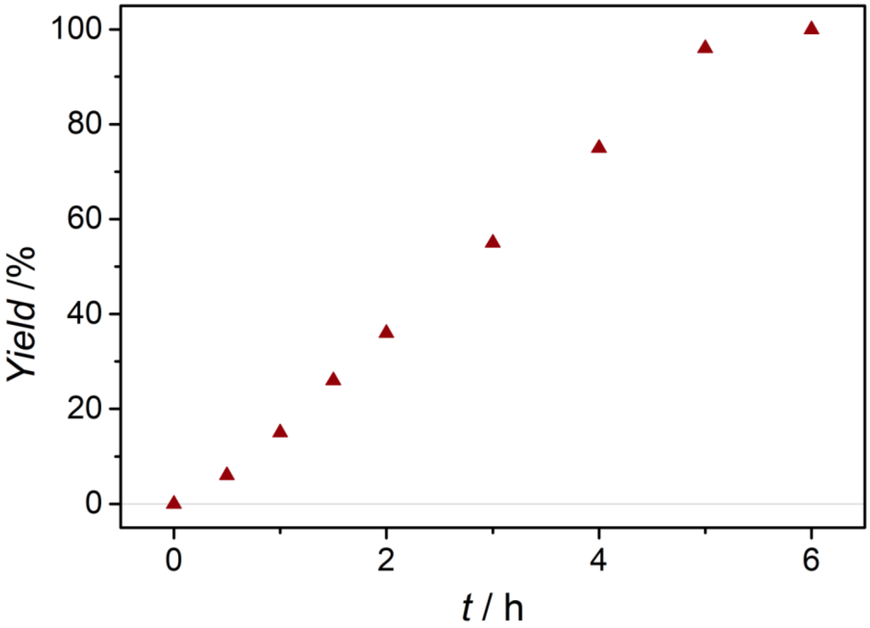

Supplementary Figure 36. Conversion vs. times plot of the photoredox catalyzed radical cation Diels-Alder cycloaddition of 100 mM ***t*An** and 500 mM **DMB** using **[Cr(tpe)$_2$]$^{3+}$** (1 mM) in CH_3CN with 460 nm light excitation.

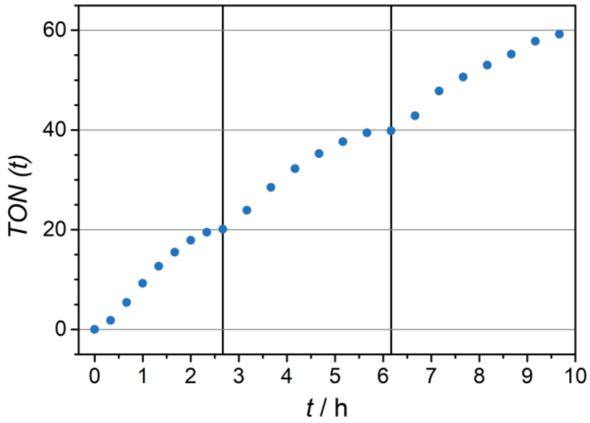

Supplementary Figure 37. Conversion vs. times plot of the photoredox catalyzed radical cation Diels-Alder cycloaddition of 100 mM ***t*An** and 500 mM **DMB** using **[Cr(tpe)$_2$]$^{3+}$** (5 mM) in CH_3CN with 460 nm light excitation. After full consumption of ***t*An**, further ***t*An** and **DMB** was added to replace the converted material (two times indicated by thick vertical lines). Due to the large number of samples withdrawn from the reaction mixture, a total volume of 4 mL was used, so that the upper part of the vial was less illuminated which accounts for the slower reaction as compared to the experiment shown in Supplementary Figure 32.

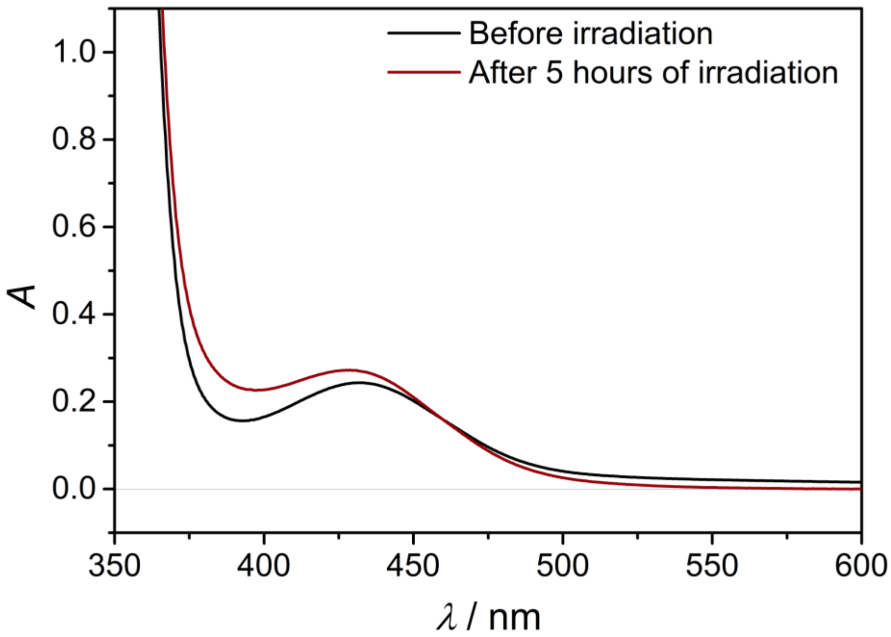

Supplementary Figure 38. UV/Vis spectra of **[Cr(tpe)$_2$][BF$_4$]$_3$** (5 mM) in aerated acetonitrile before (red) and after 5 h (black) irradiation (460 nm) in the presence of ***t*An** (100 mM) and **DMB** (500 mM). The slight blue shift of the absorption maximum might arise from the consumption of ***t*An** and thus a change in the high energy part of the spectrum.

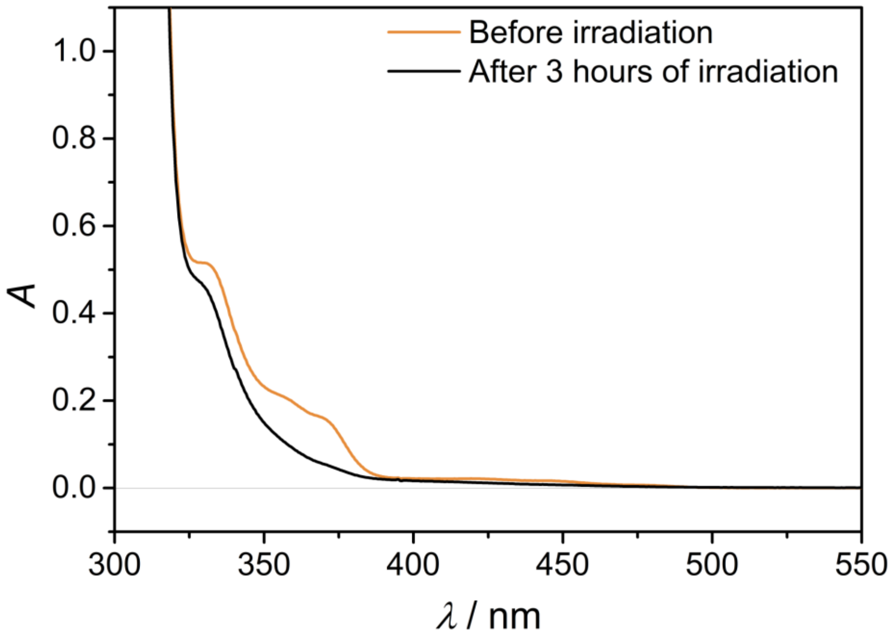

Supplementary Figure 39. UV/Vis spectra of **[Cr(dmcbpy)$_3$][BF$_4$]$_3$** (1 mM) in aerated acetonitrile before (orange) and after 3h (black) irradiation (460 nm) in the presence of ***t*An** (100 mM) and **DMB** (500 mM). The spectrum was recorded after ca. 10 fold dilution.

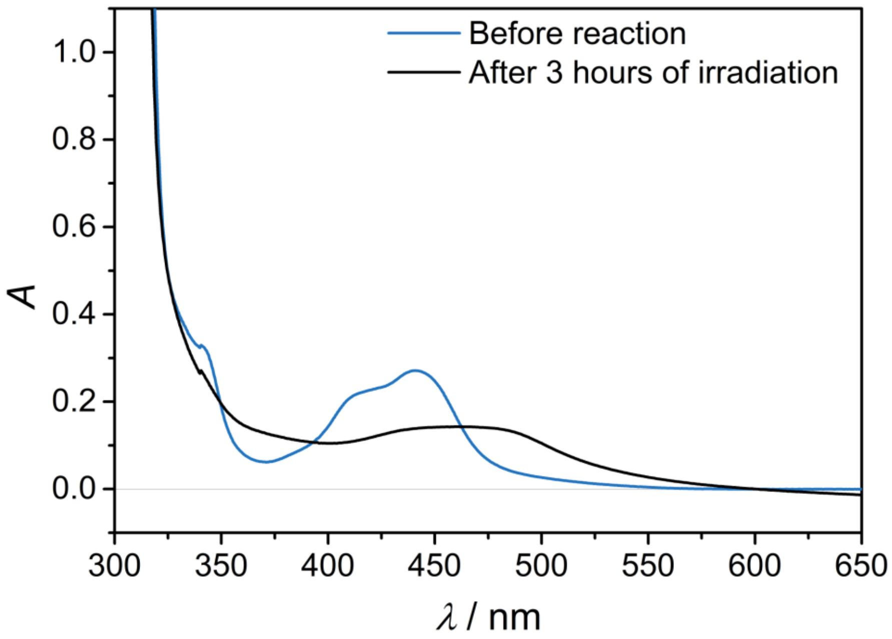

Supplementary Figure 40. UV/Vis spectra of **[Ru(bpz)$_3$][PF$_6$]$_2$** (1 mM) in aerated acetonitrile before (blue) and after 3h (black) irradiation (460 nm) in the presence of ***t*An** (100 mM) and **DMB** (500 mM). The spectrum was recorded after ca. 100 fold dilution.

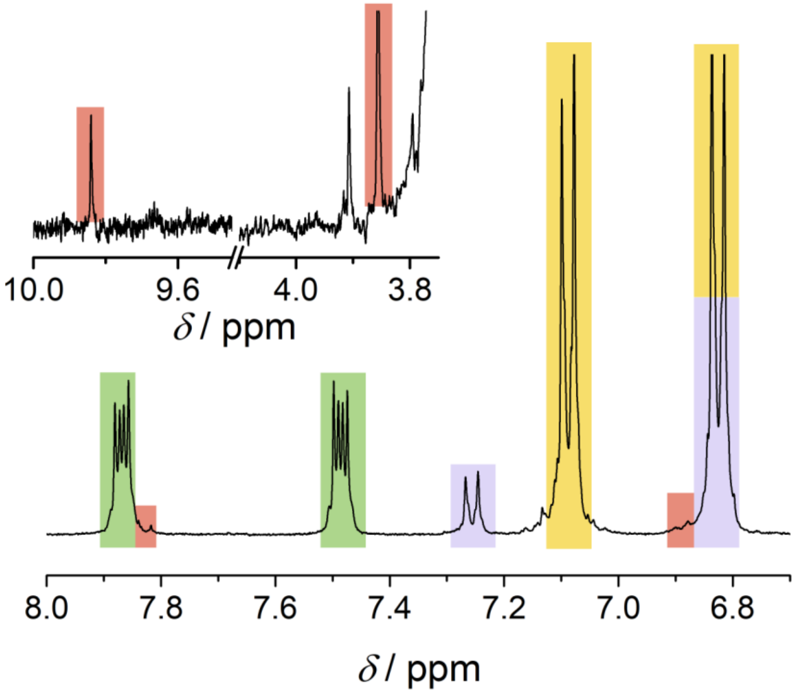

Supplementary Figure 41. Partial ^{1}H NMR spectrum of the reaction using 100 mM ***t*An** (purple), 500 mM **DMB** and 5 mM **[Cr(tpe)$_2$][BF$_4$]$_3$** in acetonitrile using naphthalene (green) as internal standard with 460 nm light excitation after 8.5 h. Product resonances of DAP highlighted in yellow. Resonances of 4-anisaldehyde highlighted in red.

References

1 Neese, F. (2012) The ORCA program system. WIREs Comput. Mol. Sci. 2, 73–78.

2 a) Becke, A. D. (1993) Density-functional thermochemistry. III. The role of exact exchange. J. Chem. Phys. 98, 5648–5652. b) Lee, C.; Yang, W.; Parr, R. G. (1988) Development of the Colle-Salvetti correlation-energy formula into a functional of the electron density. Phys. Rev. B 37, 785–789. c) Miehlich, B.; Savin, A.; Stoll, H.; Preuss., H. (1989) Results obtained with the correlation energy density functionals of becke and Lee, Yang and Parr. Chem. Phys. Lett. 157, 200–206.

3 Weigend, F.; Ahlrichs, R. (2005) Balanced basis sets of split valence, triple zeta valence and quadruple zeta valence quality for H to Rn: Design and assessment of accuracy. Phys. Chem. Chem. Phys. 7, 3297–3305.

4 Weigend, F. (2006) Accurate Coulomb-fitting basis sets for H to Rn. Phys. Chem. Chem. Phys. 8, 1057–1065.

5 Neese, F.; Wennmohs, F.; Hansen, A.; Becker, U. (200) Efficient, approximate and parallel Hartree–Fock and hybrid DFT calculations. A 'chain-of-spheres' algorithm for the Hartree–Fock exchange. Chem. Phys. 356, 98–109.

6 Izsák, R.; Neese, F. (2011) An overlap fitted chain of spheres exchange method. J. Chem. Phys. 135, 144105.

7 Pantazis, D. A.; Chen, X.-Y.; Landis, C. R.; Neese, F. (2008) All-Electron Scalar Relativistic Basis Sets for Third-Row Transition Metal Atoms. J. Chem. Theory Comput. 4, 908–919.

8 a) van Lenthe, E.; Baerends, E. J.; Snijders, J. G. (1993) Relativistic regular two-component Hamiltonians. J. Chem. Phys. 99, 4597–4610; b) van Wüllen, C. (1998) Molecular density functional calculations in the regular relativistic approximation: Method, application to coinage metal diatomics, hydrides, fluorides and chlorides, and comparison with first-order relativistic calculations. J. Chem. Phys. 109, 392–399.

9 Grimme, S.; Antony, J.; Ehrlich, S.; Krieg, H. (2010) A consistent and accurate ab initio parametrization of density functional dispersion correction (DFT-D) for the 94 elements H-Pu. J. Chem. Phys. 132, 154104.

10 Grimme, S.; Ehrlich, S.; Goerigk, L. (2011) Effect of the damping function in dispersion corrected density functional theory. J. Comput. Chem. 32, 1456–1465.

11 Barone, V.; Cossi, M. (1998) Quantum Calculation of Molecular Energies and Energy Gradients in Solution by a Conductor Solvent Model. J. Phys. Chem. A 102, 1995–2001.

12 Hasebe, N.; Suzuki, K.; Horiuchi, H.; Suzuki, H.; Yoshihara, T.; Okutsu, T.; Tobita, S. (2015) Absolute Phosphorescence Quantum Yields of Singlet Molecular Oxygen in Solution Determined Using an Integrating Sphere Instrument. Anal. Chem. 87, 2360–2366.

13 Treiling, S., Wang, C., Förster, C., Reichenauer, F., Kalmbach, J., Boden, P., Harris, J. P., Carrella, L., Rentschler, E., Resch-Genger, U., Reber, C., Seitz, M., Gerhards, M., Heinze, K. (2019) Luminescence and Light-driven Energy and Electron Transfer from an Exceptionally Long-lived Excited State of a Non-innocent Chromium(III) Complex. Angew. Chem. Int. Ed. 58, 18075-18085.
